# Per- and polyfluoroalkyl substances, gestational weight gain, postpartum weight retention and body composition in the UPSIDE cohort

**DOI:** 10.1186/s12940-023-01009-3

**Published:** 2023-09-02

**Authors:** Carolyn W. Kinkade, Zorimar Rivera-Núñez, Sally W. Thurston, Kurunthachalam Kannan, Richard K. Miller, Jessica Brunner, Eunyoung Wong, Susan Groth, Thomas G. O’Connor, Emily S. Barrett

**Affiliations:** 1 Environmental and Occupational Sciences Institute, Rutgers University, Piscataway, 170 Frelinghuysen Road, Piscataway, NJ 08854 USA; 2 Department of Biostatistics and Epidemiology, Rutgers School of Public Health, Piscataway, NJ USA; 3 Department of Biostatistics and Computational Biology, University of Rochester School of Medicine and Dentistry, Rochester, NY USA; 4 Department of Environmental Medicine, University of Rochester School of Medicine and Dentistry, Rochester, NY USA; 5 Department of Environmental Medicine, Department of Pediatrics, New York University Grossman School of Medicine, New York, NY USA; 6 Obstetrics and Gynecology, University of Rochester School of Medicine and Dentistry, Rochester, NY USA; 7 Psychiatry, University of Rochester, Rochester, NY USA; 8 School of Nursing, University of Rochester, Rochester, NY USA

**Keywords:** Gestational weight gain, Pregnancy, Per- and poly- fluoroalkyl substances, Endocrine disruption, Postpartum weight retention, Adiposity, Body mass index

## Abstract

**Background:**

Per- and polyfluoroalkyl substances (PFAS) are synthetic chemicals found in drinking water and consumer products, resulting in ubiquitous human exposure. PFAS have been linked to endocrine disruption and altered weight gain across the lifespan. A limited and inconsistent body of research suggests PFAS may impact gestational weight gain (GWG) and postpartum body mass index (BMI), which are important predictors of overall infant and maternal health, respectively.

**Methods:**

In the Understanding Pregnancy Signals and Infant Development (UPSIDE/UPSIDE-MOMs) study (*n* = 243; Rochester, NY), we examined second trimester serum PFAS (PFOS: perfluorooctanesulfonic acid, PFOA: perfluorooctanoic acid, PFNA: perfluorononanoic acid, PFHxS: perfluorohexanesulfonic acid, PFDA: perfluorodecanoic acid) in relation to GWG (kg, and weekly rate of gain) and in the postpartum, weight retention (PPWR (kg) and total body fat percentage (measured by bioelectrical impedance)). We fit multivariable linear regression models examining these outcomes in relation to log-transformed PFAS in the whole cohort as well as stratified by maternal pre-pregnancy BMI (< 25 vs. =  > 25 kg/m^2^), adjusting for demographics and lifestyle factors. We used weighted quantile sum regression to find the combined influence of the 5 PFAS on GWG, PPWR, and body fat percentage.

**Results:**

PFOA and PFHxS were inversely associated with total GWG (PFOA: ß = -1.54 kg, 95%CI: -2.79, -0.30; rate ß = -0.05 kg/week, 95%CI: -0.09, -0.01; PFHxS: ß = -1.59 kg, 95%CI: -3.39, 0.21; rate ß = -0.05 kg/week, 95%CI: -0.11, 0.01) and PPWR at 6 and 12 months (PFOA 6 months: ß = -2.39 kg, 95%CI: -4.17, -0.61; 12 months: ß = -4.02 kg, 95%CI: -6.58, -1.46; PFHxS 6 months: ß = -2.94 kg, 95%CI: -5.52, -0.35; 12 months: ß = -5.13 kg, 95%CI: -8.34, -1.93). PFOA was additionally associated with lower body fat percentage at 6 and 12 months (ß = -1.75, 95%CI: -3.17, -0.32; ß = -1.64, 95%CI: -3.43, 0.16, respectively) with stronger associations observed in participants with higher pre-pregnancy BMI. The PFAS mixture was inversely associated with weight retention at 12 months (ß = -2.030, 95%CI: -3.486, -0.573) amongst all participants.

**Conclusion:**

PFAS, in particular PFOA and PFHxS, in pregnancy are associated with altered patterns of GWG and postpartum adiposity with potential implications for fetal development and long-term maternal cardiometabolic health.

**Supplementary Information:**

The online version contains supplementary material available at 10.1186/s12940-023-01009-3.

## Background

Per- and poly-fluoroalkyl substances (PFAS) are a class of more than 9000 man-made chemicals that have been widely used in manufacturing processes and consumer products since the 1940’s [1–3]. PFAS contain at least one perfluoroalkyl group where fluorine replaces all hydrogen substituents and that covalent C-F bond makes them highly resistant to degradation, resulting in their persistence and accumulation in the environment, biota, and humans [1, 2]. In 2000, mounting evidence of PFAS’ adverse impacts on environmental and human health led to a voluntary phase out of perfluorooctanesulfonic acid (PFOS) and its precursors, compounds now referred to as legacy PFAS [4]. Concentrations of legacy PFAS in human serum have been declining for two decades since the phase out, nevertheless they are detectable in virtually 100% of individuals sampled in the United States and elsewhere [5, 6]. At present, major sources of human exposure to PFAS include consumer products (e.g., clothing, upholstery, cleaners, automobiles), drinking water, and diet [6, 7].

Extensive research has examined the potential health impacts of PFAS, including their ability to disrupt endocrine and metabolic pathways. This may be of particular concern during pregnancy, a vulnerable period during which exposure to endocrine disrupting chemicals may have short- and long-term consequences for the fetus [8–10]. Indeed, many studies have demonstrated that gestational PFAS exposure is associated with smaller size at birth and higher BMI in childhood and during adolescence [11–17]. By contrast, impacts of prenatal PFAS exposure on maternal health remain relatively understudied, despite increasing recognition that maternal health in pregnancy predicts future maternal risk of metabolic and cardiovascular disease [18–20]. Gestational weight gain (GWG) is monitored across pregnancy as part of standard clinical care and a signifier of overall pregnancy health [21]. Insufficient GWG is associated with small for gestational age and preterm birth, while excess GWG is associated with large for gestational age, macrosomia, and caesarean delivery [22, 23]. Excess GWG also affects maternal health, frequently leading to significant postpartum weight retention (PPWR) and in the long-term, increased risk of cardiometabolic disease including diabetes, stroke, and heart attack [24–27].

A small number of studies have examined PFAS exposures in relation to GWG, with mixed results (Supplementary Table 1) [28–32]. Several studies have observed associations between PFAS concentrations, most often PFOS, and higher GWG [28, 29, 31], while others have largely reported null associations [30, 32]. Associations have varied, furthermore, based on pre-pregnancy body mass index (BMI), although no clear pattern has emerged across studies [28, 29, 32]. Some studies have demonstrated stronger associations in women with lower pre-pregnancy BMI (< 25 kg/m^2^), while others have reported stronger associations in women with higher pre-pregnancy BMI (> = 25 kg/m^2^) [28, 31]. Two studies, Project Viva and the New Hampshire Birth Cohort study, have included maternal postpartum follow-up, observing that higher legacy PFAS concentrations in pregnancy were associated with increased weight retention in the years postpartum [29, 33]. The inconsistences observed across these studies is also reflected in the larger body of work on PFAS and obesity, which is characterized by conflicting results suggesting PFAS may act both obsesogenically as well as anti-obsesogenically, depending on factors including (but not limited to) biological sex and life stage as well as level of exposure [34–38].

Beyond inconsistent findings, several key limitations of the prior research on PFAS and maternal GWG and PPWR warrant further consideration. First, prior studies have not considered potentially important confounders, most notably diet, that may contribute to PFAS concentrations as well as weight measures [39]. Precision variables such as physical activity have also been largely ignored [40, 41]. Second, much of the existing literature is based on biospecimens collected 10–20 years ago when PFOS and PFOA levels were far higher than current U.S. levels. Finally, the lack of data on postpartum outcomes warrants further research given the potential importance for maternal long-term health. Therefore, to extend the limited literature on this topic to date, we use data from an ongoing, richly-phenotyped cohort to examine gestational PFAS exposure in relation to GWG across pregnancy. We additionally consider PPWR and for the first time, evaluate associations between gestational PFAS exposures and postpartum adiposity, as measured by bioelectrical impedance (BIA) at 6 and 12 months post-delivery.

## Materials and methods

### Study participants

The Understanding Pregnancy Signals and Infant Development (UPSIDE) study recruited healthy pregnant participants from participating obstetric clinics affiliated with the University of Rochester Medical Center (URMC) from 2015–2019 [42]. Participants carrying singleton fetuses were eligible to participate if they met the following criteria: (1) 18–45 years of age; (2) less than 13 weeks pregnant; (3) planned to deliver at URMC; (4) medically normal risk pregnancy (as determined by obstetric clinic staff); (5) no history of psychotic illness; (6) no heavy substance abuse; and (7) able to communicate in English. Participants completed study visits in each trimester including a blood draw. In total, 294 participants gave birth to a live infant in the study. Children delivered at term (> 37 weeks) were eligible for postnatal follow-up in UPSIDE and their mothers were invited to enroll in the ancillary UPSIDE MOMS study, which examines biological changes in the postpartum period and includes study visits at 6 and 12 months postpartum (as well as at 2, 3, and 4 years postpartum which are ongoing and therefore not included in the current analysis) [18]. The UPSIDE MOMS study was funded and started recruitment well after the first UPSIDE participants delivered their infants; as a result some mothers (*n* = 93) were not able to be approached for postnatal participation. Additionally, given our interests in weight changes in the postpartum, women who became pregnant again prior to consent were not eligible for postnatal follow-up. In total, 97% of UPSIDE participants approached enrolled in UPSIDE MOMS. Both UPSIDE and UPSIDE MOMS were approved by the URMC and Rutgers University Institutional Review Boards (IRBs) and for both projects, all participants signed informed consent at the time of enrollment. Among participants enrolled in UPSIDE MOMS, their postpartum follow-up was discontinued if they became pregnant again such that all data collected represent true postpartum measures timed relative to the original index pregnancy.

### Exposure assessment

Blood was collected at the 2^nd^ trimester visit and after processing, serum was stored at -80^0^ C. Aliquots were shipped on dry ice to the Wadsworth Center’s HHEAR Laboratory Hub (Albany, NY) [43]. The following PFAS were analyzed: perfluorobutanesulfonic acid (PFBS), perfluorohexane-1-sulfonic acid (PFHxS), PFOS, perfluoroheptanoic acid (PFHpA), PFOA, perfluorononanoic acid (PFNA), perfluorodecanoic acid (PFDA), perfluroundecanoic acid (PFUNDA), perfluorododecanoic acid (PFDODA), perfluorooctanesulfonamide (PFOSA), n-ethyl perfluorooctane sulfonamido acetic acid (NETFOSAA), n-methyl perfluorooctane sulfonamido acetic acid (NMFOSAA), perfluoro-n-pentanoic acid (PFPeA), and perfluorohexanoic acid (PFHxA). Hybrid solid-phase extraction (SPE) and ultrahigh-performance liquid chromatography-tandem mass spectrometry (UPLC-MS/MS) technique was used with an initial step to precipitate proteins and endogenous biological interferences from serum [44]. The sample was prepped by adding isotope labelled internal standards to 250 µL of serum. To this, 1% ammonium formate was added and the mixture was shaken. After centrifugation, the supernatant was quantitively transferred to a hybrid-SPE-phospholipid cartridge, and the eluate was collected for analysis [44]. Separation was carried out on an Acquity BEH C18 (1.7 um, 50 × 2.1 mm) column (Waters, Milford, MA). The UPLC mobile phase was solvent A: 100% MeOH, solvent B: 0.1% ammonium acetate in water (w/v). Electrospray triple quadrupole tandem mass spectrometry (ESI–MS/MS) (API 5500; AB SCIEX, Framingham, MA, USA) was used in multiple reaction monitoring mode under negative ionization. Isotope-dilution method was used to quantify PFAS. Daily Limit of Detection (LOD) values reported were averaged to establish the LOD for the whole study. The daily LODs were used to identify the valid values for each sample. Only PFAS with  > 80% above LOD (namely PFOS, PFOA, PFNA, PFHxS and PFDA) were included in the current analysis. For those PFAS, individual observations below the LOD were imputed as LOD/√2.

#### Outcome assessment

##### Gestational weight gain

Electronic medical records for all UPSIDE participants were abstracted by trained examiners to obtain data on the index pregnancy including maternal weight and gestational age at each clinical visit. Participant weight at the earliest first trimester clinical visit (7.4 ± 1.9 weeks) was used as a proxy for pre-pregnancy weight, a practice in pregnancy cohorts when clinically recorded preconception weight is not available [45–47]. To estimate trimester-specific weight gain, we imputed maternal weights at 14 and 28 weeks by interpolation based on the nearest recorded weights just prior to and just after 14 (mean 12.0 ± 1.4 and 16.3 ± 1.5) or 28 (mean 25.7 ± 2.2 and 29.5 ± 1.1) weeks (Supplementary Fig. 1). For participants who did not have a recorded weight in the last week prior to delivery (*n* = 44), final weight was imputed if data were available within 6 weeks prior to delivery (for *n* = 1 participant, data were not available). Total weight gain was calculated as the sum of weight gain in the first and second trimesters plus observed or imputed third trimester gain. Mid/late pregnancy weight gain (sum of 2^nd^ and 3^rd^ trimesters) is also included because of variability in gestational age at first weight collection. Rate of gain is average weekly gain per term (trimester, mid/late pregnancy, or total). In addition, in order to compare results to widely-used clinical guidelines, for each participant, we additionally determined whether total GWG was insufficient, appropriate, or excessive based on pre-pregnancy BMI using standard Institute of Medicine (IOM) recommendations for term singleton pregnancies [21].

##### Postpartum weight gain and adiposity

For the subset of UPSIDE participants (*n* = 5 participants contributed data to only the postpartum analysis, due to missing prenatal covariates) who went on to participate in UPSIDE MOMS, at 6- and 12-month postpartum visits, participant weight was recorded and body fat percentage was measured through bioelectric impedance analysis (BIA) following guidelines recommended by the manufacturer (Tanita Body Composition Analyzer MC-780, Tokyo, Japan). BIA is not recommended in pregnant women which is why body composition measurements were limited to the postpartum period. Compared to anthropometrics alone, BIA is considered a more accurate tool to assess potential risk associated with cardiometabolic health outcomes [48, 49]. PPWR was calculated as 6- or 12- month postpartum weight minus the first recorded weight in the first trimester.

##### Covariates

Covariates were selected a priori based on previous literature and a directed acyclic graph (Supplementary Fig. 2). Data on potential covariates of interest were collected from prenatal questionnaires in each trimester as well as through the medical record review and interviews at postpartum study visits. These included: fetal/infant sex, maternal age, education (high school or less, more than high school), parity (nulliparous, multiparous), and smoking during pregnancy (any). Maternal race/ethnicity (categorized as non-Hispanic White, non-Hispanic Black, and other based on the distribution of participants) was included as a proxy for structural racism and injustice that may contribute to differential exposures to PFAS as well as overweight and obesity [50–52]. Early pregnancy BMI (kg/m^2^) was calculated from prenatal maternal height and the first weight collected in early pregnancy. We refer to BMI < 25 (including underweight and normal) as ‘lower’ and BMI >  = 25 (overweight and obese) as ‘higher’. Physical activity was assessed in each trimester as well as at 6 and 12 months postpartum using the validated Pregnancy Physical Activity Questionnaire (PPAQ), from which total weekly metabolic equivalents (metabolic equivalents of task (METs)) were calculated [53]. For prenatal models (2^nd^, 3^rd^ trimesters and total GWG), we averaged second and third trimester METs, while postnatal models used postnatal METs at the relevant outcome timepoint (6 or 12 months). Maternal diet was assessed in mid-late pregnancy and at the 6-month postnatal visit by a trained nutritionist using standardized 24-h dietary recall approach and based on that, total kilocalories was calculated [54, 55]. Current breastfeeding (yes/no) was recorded at each postnatal visit based on maternal self-report.

### Statistical analysis

Descriptive statistics for the cohort were calculated including mean, standard deviation, percentage, and range. PFAS were not normally distributed so log-transformed PFAS were used for bivariate analyses and multivariable linear regression models. Spearman-rank coefficients were calculated to examine PFAS concentrations in relation to each other, and to trimester specific and total GWG, PPWR and body fat percentage at 6 and 12 months.

#### Gestational weight gain models

We fit linear regression models (unadjusted and adjusted) to assess the association between PFAS exposures and trimester-specific, mid/late pregnancy, and total gestational weight gain, as well as rate of gain in each term. Covariates retained in final models were selected based on directed acyclic graphs and if they changed effect estimates by 10%. We used a staged modeling approach. Minimally adjusted models include these covariates: maternal race/ethnicity, education, parity, age, early pregnancy BMI, and smoking. Fully adjusted models for trimester 2, trimester 3, and rate (kg/week) include minimally adjusted covariates and: gestational age at serum collection, mid-late pregnancy kcal/day and METs/week. Fully adjusted models for mid/late and total GWG additionally include gestational age at delivery. Per interquartile range (IQR) results are fully adjusted models scaled to one IQR increase in log-transformed PFAS. In sensitivity analyses, we refit total GWG models excluding preterm births (*n* = 13). In light of prior literature demonstrating the importance of pre-pregnancy BMI in associations between PFAS concentrations and GWG, we additionally examined effect modification by early pregnancy BMI (< 25 vs. ≥ 25 kg/m^2^) [28, 30]. We did so by first fitting stratified models and secondarily, fitting models in the full cohort that included interaction terms for early pregnancy BMI group and each individual PFAS. We report fully adjusted models in the main text and figures.

#### Postpartum endpoint models

To examine associations with postpartum endpoints, we fitted a second set of models examining PFAS in relation to PPWR and adiposity at 6 and 12 months. Based on the same covariate selection process described earlier we created staged models. Minimally adjusted models include these covariates: race/ethnicity, education, maternal age, parity, pre-pregnancy BMI. Fully adjusted includes minimally adjusted covariates and energy intake (kcal/day at 6 m), physical activity (METs/week at 6 or 12 months), gestational age at serum sampling, gestational age at delivery, breastfeeding (yes/no) and weeks post-partum as covariates. Fully adjusted models are reported in the text and figures.

#### Mixtures analysis

To supplement our main analyses and examine the joint impacts of multiple PFAS on these outcomes, weighted quantile sum regression method was applied to test the influence of an exposure mixture of PFOS, PFOA, PFNA, PFHxS, and PFDA on average rate of gain in mid/late pregnancy and postpartum outcomes [56]. We used 1000 bootstraps and ran the models for negative then positive associations. Models where less than 10% of bootstrapped weights were associated with the mixture in a given direction are marked in the tables, and we recommend interpreting those results with caution. We used the same covariates fitted for the multivariable linear models for consistency and fitted models with the whole cohort as well as stratified by early pregnancy BMI. For gestational weight gain, we selected one endpoint, rate of gain across second and third trimesters, to reduce the number of comparisons. For the postpartum period, we evaluated the same endpoints used in linear regression models.

Data analysis was performed in R studio (Version 4.1.0). Mixtures analysis was conducted using gWQS (version 3.0.4) package.

## Results

Of 294 births in the study, 286 participants had 2^nd^ trimester serum samples analyzed for PFAS and of those, 243 participants had complete covariates for the prenatal phase of the current analysis (Table 1). The mean age at study enrollment was 29.3 ± 4.4 years. Over half (53%) of the cohort was overweight or obese at enrollment. Most participants had a prior birth (66.7%), were non-Hispanic white (62.1%), and had more than a high school education (67.9%). Prenatal energy intake was 2157.6 ± 311.8 kcal/day and prenatal physical activity was 341.5 ± 173.9 METs/week. Only a small fraction of participants included in the prenatal analysis smoked during pregnancy (6.2%) and preterm birth was similarly rare (5.3%). The distribution of covariates among those included in the prenatal sample were generally representative of the UPSIDE cohort as a whole (not shown).

**Table 1:** Descriptive statistics of UPSIDE participants included in the current analysis

	**Participants contributing prenatal data (*n* = 243)**	**Participants contributing prenatal and postpartum data (*n* = 139)**^**a**^
**Demographic and lifestyle measures- continuous**	**Mean (SD)**	
Age (years)	29.3 (4.4)	30.0 (4.2)
Pre-pregnancy BMI (kg/m^2^)	27.9 (7.2)	27.1 (6.8)
Gestational age at delivery (wks)	39.5 (1.4)	39.7 (1.2)
Gestational age at PFAS measurement (wks)	21.1 (1.8)	21.1 (1.6)
Prenatal Energy Intake (kcal/day)	2157.6 (311.8)	2173.5 (323.3)
Postpartum Energy Intake (kcal/day)	-	1946.1 (408.6)
Prenatal Physical Activity (METs/day)	341.5 (173.9)	338.8 (171.2)
Postpartum Physical Activity (METs/week) – 6 months	-	371.4 (158.2)
Postpartum Physical Activity (METs/week) – 12 months	-	391.5 (199.0)
Infant birthweight (grams)	3386.6 (520.2)	3464.4 (474.7)
**Demographic and lifestyle measures- categorical**	**n (%)**	
Preterm birth	13 (5.3)	0
Race/Ethnicity		
Non-Hispanic White	151 (62.1)	96 (69.1)
Non-Hispanic Black	51 (21.0)	27 (19.4)
Other	41 (16.9)	16 (11.5)
Parity		
Nulliparous	81 (33.3)	45 (32.4)
Parous	162 (66.7)	94 (67.6)
Education		
HS or less	78 (32.1)	34 (24.5)
More than HS	165 (67.9)	105 (75.5)
Smoking during Pregnancy (any)	15 (6.2)	7 (5.0)
Infant sex (male)	124 (51.0)	74 (53.2)
Breastfeeding (any) – 6 months	-	87 (70.2)
Breastfeeding (any) –12 months	-	60 (57.7)
**Gestational weight gain measures (in kg)**	**Mean (SD)**	
1^st^ Trimester	0.69 (2.29)	0.66 (2.35)
Average weekly – 1^st^ Trimester (kg/week)	0.13 (0.34)	0.13 (0.35)
2^nd^ Trimester	6.26 (3.22)	6.51 (3.06)
Average weekly – 2 ^nd^ Trimester (kg/week)	0.45 (0.23)	0.47 (0.22)
3^rd^ Trimester	5.27 (3.06)	5.47 (2.95)
Average weekly – 3 ^rd^ Trimester(kg/week)	0.48 (0.28)	0.49 (0.25)
Mid/Late	11.53 (5.29)	12.06 (4.96)
Average weekly – Mid/Late (kg/week)	0.46 (0.21)	0.48 (0.19)
Total	12.22 (6.27)	12.73 (6.05)
Average weekly – across pregnancy (kg/week)	0.39 (0.20)	0.40 (0.19)
**GWG by IOM recommendations**	**n (%)**	
Less than IOM recommended	50 (20.6)	24 (18.0)
IOM recommended	97 (39.9)	56 (42.1)
Greater than IOM recommended	96 (39.5)	53 (39.8)
**Postpartum weight retention and adiposity**	**Mean (SD)**	
Weeks since delivery – 6 months	-	27.6 (3.8)
Weeks since delivery – 12 months	-	55.4 (5.6)
Weight retention (kg) – 6 months	-	0.98 (5.35)
Weight retention (kg) – 12 months	-	-0.02 (6.47)
Body fat (%) – 6 months	-	31.9 (8.3)
Body fat (%) – 12 months	-	31.8 (8.9)

*Abbreviations: BMI* body mass index, *GWG* gestational weight gain, *HS* high school, *IOM* Institute of Medicine, *kcal* kilocalories, *kg* kilograms, *MET* metabolic equivalent of task, *PFAS* per- and poly-fluoroalkyl substances

^a^*N* = 5 participants contributed data only to the postpartum analysis. The number of participants evaluated at 6 and 12 months postpartum was 124 and 104, respectively

Compared to participants with only prenatal data, participants who continued into the postpartum phase of the study were slightly older on average (30.0 ± 4.2 years) and were predominantly non-Hispanic White (70.7%) with greater than a high school education (75.9%). Early pregnancy BMI was lower among women who contributed postnatal data (27.1 ± 6.8 kg/m^2^) compared to those only contributing prenatal data (29.2 ± 7.7 kg/m^2^, Supplementary Table 2). Most participants reported breastfeeding at 6 (70.2%) and 12 (57.7%) months. Compared to the prenatal period, on average, energy intake was lower (1946.1 ± 408.6 kcal/day at 6 months) and physical activity was higher (371.4 ± 158.2 and 391.5 ± 199.0 at 6 and 12 months postpartum, respectively).

On average, the mid-pregnancy blood draw for PFAS measurement occurred at 21.1 ± 1.8 weeks gestational age. PFOS, PFOA, PFNA, and PFHxS were detected in 100% of serum samples with PFOS present at the highest concentrations (median: 2.52 ng/ml, range: 0.61–21.3 ng/ml) (Table 2). PFDA was detected in 83% of samples (median: 0.08 ng/ml, range: 0.01–0.68 ng/ml). PFAS concentrations were comparable between (1) participants who only participated prenatally and those who participated in both study phases (Table 2) and (2) participants included in the postnatal period and those lost to follow up (Supplementary Table 2). PFAS were moderately correlated with one another in the whole cohort (Supplementary Table 3), as well as when stratified by lower and higher pre-pregnancy BMI groups (not shown).

**Table 2:** PFAS concentrations (ng/ml) in UPSIDE participants included in the current analysis^a^

PFAS	N	LOD	% < LOD	Mean	STD	50^th^	90^th^	95^th^	Min	Max
*Participants contributing prenatal data*										
PFOS	243	0.02	0	2.86	2.01	2.52	4.28	5.51	0.61	21.3
PFOA	243	0.02	0	0.68	0.42	0.59	1.16	1.46	0.04	2.74
PFNA	243	0.032	0	0.31	0.30	0.25	0.49	0.62	0.06	2.87
PFHxS	243	0.02	0	1.90	0.87	1.74	3.03	3.48	0.56	7.17
PFDA	243	0.02	17	0.08	0.08	0.06	0.15	0.20	0.01	0.68
*Participants contributing postnatal data*										
PFOS	139	0.02	0	2.94	2.41	2.43	4.18	6.56	0.61	21.3
PFOA	139	0.02	0	0.70	0.57	0.58	1.09	1.39	0.04	5.45
PFNA	139	0.032	0	0.34	0.39	0.23	0.47	0.93	0.06	2.87
PFHxS	139	0.02	0	1.93	0.81	1.75	3.03	3.37	0.56	6.03
PFDA	139	0.02	19	0.09	0.13	0.05	0.16	0.20	0.01	1.04

*Abbr*: *LOD* Limit of Detection, *Min* Minimum, *Max* Maximum, *PFAS* per- and poly-fluoroalkyl substances, *PFDA* perfluorodecanoic acid, *PFHxS* perfluorohexanesulfonic acid, *PFNA* perfluorononanoic acid, *PFOA* perfluorooctanoic acid, *PFOS* perfluorooctanesulfonic acid, *STD* Standard Deviation

^a^*N* = 5 participants contributed data only to the postpartum analysis

On average, participants gained 12.22 ± 6.27 kg total across pregnancy, including 0.69 ± 2.29, 6.26 ± 3.22, and 5.27 ± 3.06 kg on average in the first, second, and third trimesters, respectively (Table 1). Participants with lower early pregnancy BMI gained more weight than participants with higher early pregnancy BMI overall (14.52 ± 4.25 versus 10.18 ± 7.03 kg) as well as in each individual trimester (Supplementary Table 4). Fewer than half of all participants gained weight within the IOM recommended range (39.9%) based on their early pregnancy BMI, with approximately the same percentage (39.5%) gaining more weight than recommended. Appropriate weight gain (based on IOM classifications) was more common among participants in the lower vs. higher early pregnancy BMI group (54.4% vs. 27.1%). (Supplementary Table 4). Weight gain was similar between participants recruited to UPSIDE and those included in the prenatal analysis (Supplementary Table 2).

Post-partum analyses included 124 participants contributing data at 6 months and 104 at 12 months. PPWR and body fat percentage were analyzed at 6 and 12 months (mean 27.6 ± 3.8 and 55.4 ± 5.6 weeks post-delivery, respectively; Table 1). At 6- and 12-months post-delivery participants retained 0.98 ± 5.35 and -0.02 ± 6.47 kg, respectively, compared to their early pregnancy weights. Body fat percentage was similar at 6- (31.9 ± 8.3%) and 12-months (31.8 ± 8.9%) post-delivery. PPWR was highly correlated between timepoints (*ρ* = 0.84, *p* < 0.001) and moderately correlated with body fat percentage (6 month: *ρ* = 0.47, *p* < 0.001, 12 month: *ρ* = 0.46, *p* < 0.001).

Spearman correlation coefficients between log-transformed PFAS and trimester specific and total gestational weight gain were weak (Table 3). Among women who began pregnancy with lower BMI, inverse correlations between all PFAS and weight gain were observed (range ρ: -0.002 to -0.25). By contrast, in women who started pregnancy with higher BMI, mostly weak positive correlations were observed for example total GWG (*ρ* = 0.22, *p* < 0.05)).

**Table 3:** Spearman correlation between log-transformed PFAS (ng/ml) and gestational weight gain (kg) in the UPSIDE cohort

Group	Term	PFOS	PFOA	PFNA	PFHxS	PFDA
**All participants (*n* = 243)**	Trimester 2 GWG	0.09	-0.00	-0.00	0.13*	0.11
	Trimester 3 GWG	0.01	-0.02	0.01	-0.03	0.08
	Mid/late GWG	0.06	-0.02	0.01	0.06	0.11
	Total GWG	0.09	-0.00	0.03	0.08	0.14*
**Participants with pre-pregnancy BMI < 25 kg/m**^**2**^** (*n* = 114)**	Trimester 2 GWG	-0.13	-0.14	-0.25**	-0.00	-0.20*
	Trimester 3 GWG	-0.14	-0.03	-0.09	-0.14	-0.11
	Mid/late GWG	-0.17	-0.10	-0.22	-0.09	-0.19*
	Total GWG	-0.20*	-0.15	-0.25**	-0.13	-0.20*
**Participants with pre-pregnancy BMI > = 25 kg/m**^**2**^** (*n* = 129)**	Trimester 2 GWG	0.14	0.02	0.12	0.03	0.16
	Trimester 3 GWG	0.07	-0.04	0.06	-0.02	0.16
	Mid/late GWG	0.12	-0.01	0.10	0.01	0.19*
	Total GWG	0.16	0.03	0.15	0.02	0.22*

*Abbr*: *BMI* Body Mass Index, *GWG* gestational weight gain, *PFAS* per- and poly-fluoroalkyl substances, *PFDA* perfluorodecanoic acid, *PFHxS* perfluorohexanesulfonic acid, *PFNA* perfluorononanoic acid, *PFOA* perfluorooctanoic acid, *PFOS* perfluorooctanesulfonic acid

^*^indicates *p* < 0.05

^**^indicates *p* < 0.01

**Fig. 1:**
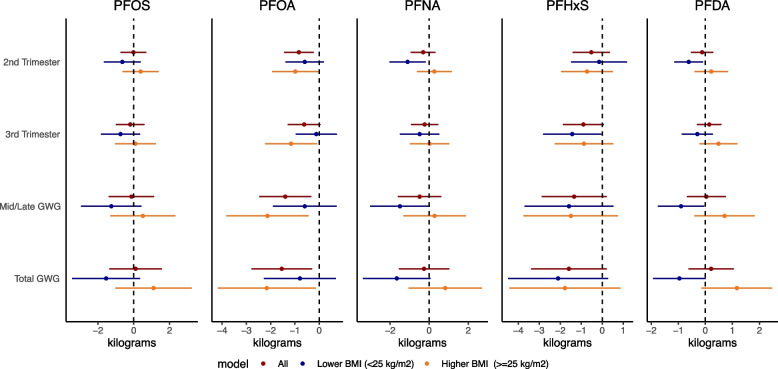
Multivariable linear models examining PFAS in relation to gestational weight gain in the UPSIDE cohort† BMI: Body Mass Index; PFAS: per- and poly-fluoroalkyl substances, PFDA: perfluorodecanoic acid; PFHxS: perfluorohexanesulfonic acid, PFNA: perfluorononanoic acid; PFOA: perfluorooctanoic acid; PFOS: perfluorooctanesulfonic acid. † The unit for PFAS concentrations is log(ng/ml) and weight gain is in kilograms. Fully adjusted models included PFAS serum weeks, smoking, parity, maternal age, education, race/ethnicity. Models considering the whole cohort include early pregnancy BMI. In addition, models examining 2^nd^ and 3^rd^ trimester weight gain, mid/late (2^nd^ + 3^rd^ trimester) and total GWG included mid-late pregnancy kcal/day and METs/week. Models examining total GWG additionally included gestational age at delivery. In models considering all participants, the n is 243. In stratified analysis, the groups are lower BMI (< 25 kg/m^2^; *n* = 114) and higher BMI (> = 25 kg/m^2^: *n* = 129)

In multivariable models examining associations between PFAS and GWG in the whole cohort, higher concentrations of PFOA and PFHxS were associated with lower GWG (Fig. 1, Supplemental Table 5). PFOA was inversely associated with weight gain in mid to late pregnancy and total GWG (2^nd^ trimester: β = -0.84 kg, 95% CI: -1.54, -0.23, 3^rd^ trimester: β = -0.62 kg, 95% CI: -1.30, 0.05, mid/late: β = -1.40 kg, 95% CI: -2.47, -0.33, mid/late per IQR: β = -1.89 kg, 95% CI: -3.34, -0.45, total: β = -1.54 kg, 95% CI: -2.79, -0.03). PFHxS was associated with lower weight gain in trimester 3 (β = -0.90 kg, 95% CI: -1.87, 0.08) as well as lower total GWG (β = -1.59 kg, 95% CI: -3.39, 0.21). PFOS, PFNA, and PFDA were not associated with total gestational weight gain in models considering the whole cohort.

In analyses stratified by maternal early pregnancy BMI, associations between log-transformed PFAS and gestational weight gain varied by early pregnancy BMI and time period (Fig. 1, Supplemental Table 5). Among women who started pregnancy with low-normal BMI, all individual PFAS were associated with lower trimester-specific and total GWG. The strongest associations in this group were observed for PFNA (β = -1.66 kg, 95% CI: -3.39, 0.07) and PFHxS (β = -2.10 kg, 95% CI: -4.48, 0.28). Although associations were strongest for total GWG, associations with trimester-specific GWG were also consistently inverse in the lower BMI group. For example, PFNA was associated with lower GWG in trimesters 2 (β = -1.11 kg; 95% CI: -2.03, -0.18) and PFHxS in trimester 3 (β = -1.43 kg; 95% CI: -2.82, -0.04). Associations among women with higher early pregnancy BMI  >  = 25 kg/m^2^ (*n* = 129) were less consistent (Fig. 1, Supplemental Table 5). In that group, PFOA was associated with lower total GWG (β = -2.16 kg; 95%: -4.17, -0.15, rate β = -0.07 kg/week; 95%: -0.14, -0.01) as well as lower GWG in the 2^nd^ (β = -0.99 kg; 95%: -1.94, -0.04) and 3^rd^ (β = -1.16 kg; 95%: -2.23, -0.09) trimesters.

In sensitivity analyses excluding women who went on to deliver preterm (*n* = 13), the magnitude and directions of associations between PFAS and total GWG were consistent with models including all participants (Supplementary Table 6). In secondary models examining PFAS*BMI interactions (rather than stratifying), the interaction term was only statistically significant for PFOS in relation to total GWG (*p* = 0.03; Supplementary Table 7).

**Fig. 2:**
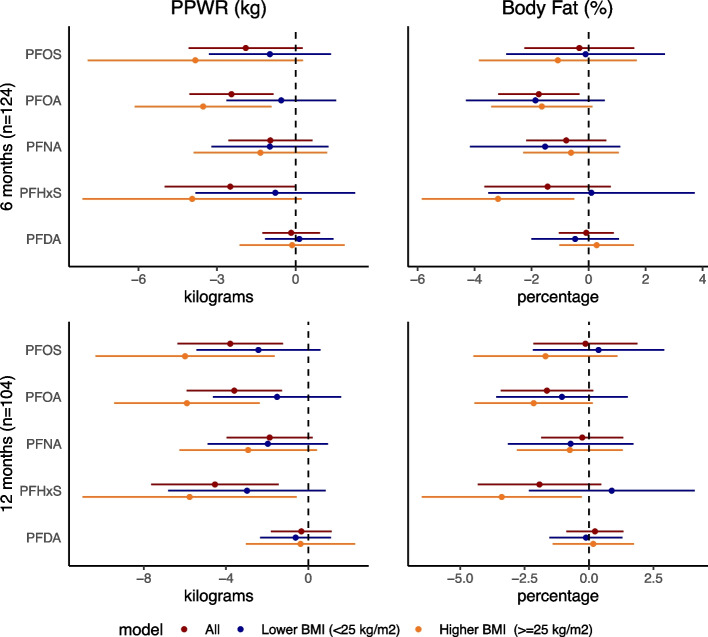
Multivariable linear models examining PFAS in relation to post-partum weight retention and total body fat^†^ Abbr: BMI: Body Mass Index; PFAS: per- and poly-fluoroalkyl substances, PFDA: perfluorodecanoic acid; PFHxS: perfluorohexanesulfonic acid, PFNA: perfluorononanoic acid; PFOA: perfluorooctanoic acid; PFOS: perfluorooctanesulfonic acid; PPWR: postpartum weight retention. † The unit for PFAS concentrations is log(ng/ml) and weight retention is in kilograms. Fully adjusted models are adjusted for race/ethnicity, education, maternal age, parity, early pregnancy BMI, and energy intake (kcal/day at 6 m), physical activity (METs/week at 6 or 12 months), gestational age at PFAS sampling, gestational age at delivery, breastfeeding (yes/no) and weeks post-partum as covariates. In 6 month models considering all participants, the n is 124 and 104 at 12 months. In stratified analysis, the groups are lower early pregnancy BMI (< 25 kg/m^2^; *n* = 66 at 6 month and 52 at 12 months) and higher early pregnancy BMI (> = 25 kg/m^2^: *n* = 58 at 6 months and 52 at 12 months)

Correlations between PFAS and PPWR and body fat percentage tended to be inverse and stronger than the correlations between PFAS and GWG measures (Supplemental Table 8). For example, among the whole cohort, PFOA was inversely related to PPWR at 6 and 12 months (6 months *ρ* = -0.20, *p* < 0.05; 12 months *ρ* = -0.25, *p* < 0.01). PFHxS was correlated with lower PPWR and body fat at 6 (PPWR: *ρ* = -0.23; body fat %: -0.19) and 12 (PPWR: ρ = -0.32; body fat %: -0.31) months. In adjusted multivariable linear models including the whole cohort, overall, higher PFAS concentrations tended to be associated with lower PPWR at 6 and 12 months post-delivery, with the strongest associations (in kg) observed for PFOA (6 months β: -2.39 95%CI -4.17, -0.61; 12 months β: -4.02; 95%CI -6.58, -1.46) and PFHxS (6 months β: -2.94; 95%CI -5.52, -0.35; 12 months β: -5.13 95%CI -8.34, -1.93; Fig. 2, Supplementary Table 9). PFOA was additionally associated with lower body fat percentage at 6 months post-delivery (β: -1.75; 95%CI: -3.17, -0.32; per IQR β: -2.36; 95%CI: -4.28, -0.43, Supplementary Table 10). In analyses stratified by early pregnancy BMI, associations tended to be more strongly inverse in the higher BMI group compared to the lower BMI group (Fig. 2, Supplementary Table 9), although interaction terms were not statistically significant (Supplementary Table 7).

**Fig. 3:**
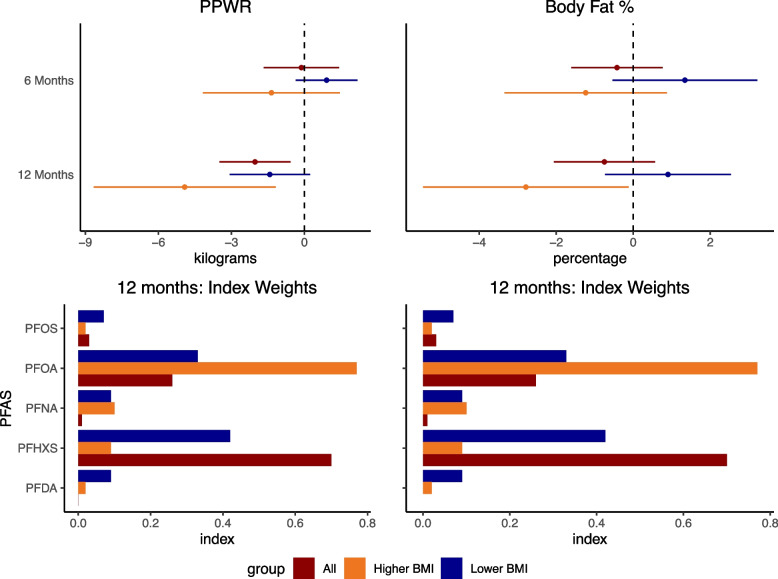
Association between weighted quantile sum of PFAS exposure and weight retention and body fat percentage at 6 and 12 months postpartum ^†^ Abbr: BMI: Body Mass Index; PFAS: per- and poly-fluoroalkyl substances, PFDA: perfluorodecanoic acid; PFHxS: perfluorohexanesulfonic acid, PFNA: perfluorononanoic acid; PFOA: perfluorooctanoic acid; PFOS: perfluorooctanesulfonic acid; PPWR: postpartum weight retention. † The unit for PFAS concentrations is log(ng/ml) and weight retention is in kilograms. Models and index weights for negative mixtures are presented. The model is adjusted for maternal race/ethnicity, education, maternal age, parity, early pregnancy BMI, energy intake (kcal/day at 6 m), physical activity (METs/week at 6 or 12 months), gestational age at PFAS sampling, gestational age at delivery, breastfeeding (yes/no) and weeks post-partum. In 6 month models considering all participants, the n is 124 and 104 at 12 months. In stratified analysis, the groups are lower early pregnancy BMI (< 25 kg/m^2^; *n* = 66 at 6 month and 52 at 12 months) and higher early pregnancy BMI (> = 25 kg/m^2^: *n* = 58 at 6 months and 52 at 12 months)

In a post hoc analysis, WQS regression focused on a smaller set of prioritized outcome measures. In women who began pregnancy with lower BMI, the exposure mixture was inversely associated with mid/late pregnancy rate of gain in both the minimally adjusted (negative WQS ß = -0.034 kg/week, 95%CI: -0.07, 0.00, positive WQS ß = -0.05 kg/week, 95%CI: -0.08, -0.01) and fully adjusted models (negative WQS ß = -0.03 kg/week, 95%CI: -0.07, 0.00, positive WQS ß = -0.05 kg/week, 95%CI: -0.08, -0.01, Supplementary Table 12), with PFHxS/PFOA most heavily weighted in the negative WQS mixture and PFOS/PFDA in the positive mixture (Supplementary Table 13). In postpartum analyses, we did not observe any associations with endpoints measured at 6 months, however we identified a PFAS mixture that was inversely associated with weight retention at 12 months in the negative minimally adjusted (ß = -1.52 kg; 95%CI: -3.10, 0.05) and fully adjusted (ß = -1.79 kg; 95%CI: -3.48, -0.10) models (Fig. 3). While estimates were in the same direction for both BMI categories, results were stronger and only statistically significant for women with BMI > 25 kg/m^2^ in early pregnancy (minimally adjusted: ß = -3.95 kg (95%CI: -6.95, -0.95); fully adjusted: ß = -4.92 kg (95%CI: -8.66, 1.18). Although we observed some indication of mixtures associated with lower body fat percentage (Fig. 3, Supplementary Table 12), these results should be viewed with caution as less than 100 bootstraps were negative (out of 1000). For postpartum outcomes with negative WQS beta estimates, PFOA and PFHxS were the most strongly weighted PFAS in the mixtures (Fig. 3).

## Discussion

In this contemporary U.S. cohort, overall, prenatal maternal PFOA and PFHxS concentrations were associated with lower mid-to-late pregnancy GWG and total GWG. Prenatal PFOS, PFOA, and PFHxS were associated with lower PPWR and adiposity in the first year postpartum. We additionally observed that associations with lower GWG tended to be stronger in participants who started pregnancy with low-normal BMI, whereas associations with postpartum endpoints were stronger in participants with higher early pregnancy BMIs. To the best of our knowledge, this is the first study to report on changes in postpartum body fat in relation to PFAS. Specifically we found that high PFOA during pregnancy was associated with lower body fat percentage at 6 months. Additionally, results of a limited set of mixtures analyses were largely consistent with primary models focused on individual metabolites.

Systematic reviews evaluating human and animal studies have concluded that maternal serum PFOA is associated with lower birthweight [57–59] with some evidence suggesting that PFAS exposure may lead to lower birthweight via disruption of the placenta [60]. GWG, a composite measure of fetal weight, placenta, water retention, fat, mammary tissue, is a widely utilized metric of pregnancy health [21, 25]. Despite ample evidence that maternal gestational weight gain has a strong impact on birth size [12–14], to date only six studies (including this one) have investigated the hypothesis that PFAS exposures in pregnancy may alter maternal GWG, with mixed results. In contrast to our results, several studies observed weak positive associations (Supplementary Table 1). In the Canadian MIREC study (*n* = 1036; 2008–2011), a weak direct association between first trimester maternal serum PFOS and GWG (ß = 0.39 kg (log2 transformed), 95%CI: 0.02, 0.75) was observed among women who started pregnancy with normal BMI, but not women who began pregnancy overweight or obese [28]. Similarly, in the U.S. LIFE study (*n* = 218; 2005–2007), pre-conception PFOS concentrations were associated with greater GWG (assessed as area under the curve) among lower BMI women (< 25 kg/m^2^) (ß = 280.29 (95%CI: 13.71, 546.86), but not higher BMI (> 25 kg/m^2^) women [32]. Romano et al. (2021) observed weak positive associations between serum concentrations of PFOA, PFOS and PFNA and GWG in the HOME study (*n* = 277; 2003–2006), however in contrast to the other studies, associations were limited to overweight and obese women (PFNA (log2 transformed) ß = 2.6 lbs, 95%CI: -0.8, 6.0) [30]. Romano also reported inverse associations between PFNA and PFHxS and GWG for women starting pregnancy with normal BMI, though the results were not significant [30]. Finally, in the AVON Study (*n* = 14,451; 1991–1992), investigators observed mostly null associations between PFAS and absolute GWG [31]. Notably, in the prior literature, positive associations between PFAS and GWG were small in magnitude, (i.e. in MIREC, ß(PFOS) = 0.39 kg (log2 transformed), 95%CI: 0.02, 0.75) or null, whereas UPSIDE found robust inverse associations per interquartile range increase for PFOA and total gestational weight gain ß = -3.28 kg, 95%CI: -5.89, -0.66, Supplementary Table 6) amongst women with higher BMI at the start of pregnancy.

Several critical factors differ between the UPSIDE cohort and prior studies (Supplementary Table 1) which may contribute to the differing results regarding the association between PFAS exposures and GWG. First, biospecimen collection for all previous studies ranged from 1991- 2011 [31, 61] and PFAS concentrations in the U.S. have dropped considerably over the same time period [62]. Notably, PFOS, PFOA, PFNA, and PFDA concentrations (median 2.9, 0.7, 0.3, 0.08 ng/ml, respectively) in the UPSIDE cohort collected from 2016–2019 were below 2017–2018 NHANES values for adult females, though PFHxS (1.9 ng/ml) was higher [63]. Project VIVA, LIFE, and the AVON cohorts reported median PFOS levels 25.7, 14.8, 13.8 ng/ml, respectively, which are up to an order of magnitude greater than the concentrations in our study (Supplementary Table 1). This is important given that the prior literature may not reflect exposures in contemporary populations, which is particularly relevant in light of evidence that some endocrine disruptors (including PFAS) may exert non-monotonic effects [17, 64–66]. Second, in addition to the earlier period of recruitment, prior literature included models only adjusted for demographics, and did not examine lifestyle factors such as physical activity and diet, which are important predictors of GWG [41, 67, 68]. By fitting both fully adjusted models including these factors and minimally adjusted models excluding them, we were able to show that results were largely consistent with and without the inclusion of these covariates. Importantly, studies that found positive associations between PFAS and GWG, as in both MIREC and AVON, the sample was predominantly White (> 80% and > 90%, respectively). UPSIDE is somewhat more diverse, with 21% of participants Non-Hispanic Black and 17% other races and ethnicities including Asian, Hispanic, Pacific Islander, and mixed race. Third, of all studies on this topic the UPSIDE cohort had the largest proportion of participants starting pregnancy overweight or obese (53%). Higher BMI participants gained less weight than low BMI participants so UPSIDE may be better suited for detecting inadequate gain. Fourth, some previous studies relied on self-report pre-pregnancy weight [29, 32]. Self-reported weights may be biased towards under-report and contribute to an overestimate of GWG. UPSIDE uses early pregnancy weight from the medical record and imputation of final weight at delivery to more accurately estimate GWG. Finally, timing of exposure assessment varied considerably between studies, ranging from pre-pregnancy to mid-pregnancy. As plasma volume and glomerular filtration rate vary across pregnancy, timing differences may also contribute to results. In summary, cohort diversity, BMI profile of the cohort, temporal trends in exposure, and timing of exposure assessment may have contributed to differences in findings between these results and the prior literature.

Few studies have examined PFAS and PPWR and of those, all have relied on weight and BMI, with no measurement of body fat, which is arguably a better predictor of postpartum health and cardiometabolic outcomes [69]. Body fat percentage is more accurate than BMI alone because (1) individuals with more lean mass may be misclassified by BMI cutoffs [70] (2) body fat percentage outperforms BMI in predicting cardiovascular disease [71], (3) high body fat percentage can occur in low BMI individuals, so BMI cutoffs are not useful as screening tools for disease interventions [55, 72]. Two studies to date have investigated PFAS associated postpartum weight outcomes among mothers [29, 33]. In Project VIVA (*n* = 1614; 1999–2002), no significant associations between PFAS (log2 transformed) and GWG were observed with the exception of a weak positive association for EtFOSAA (ß = 0.37 kg, 95%CI: 0.11, 0.62) [29]. Subsequent postpartum follow-up of that cohort indicated that prenatal PFAS concentrations were associated with increased weight retention at 1- and 3- years year post-partum (ß = 0.55 kg (95%CI: 0.07, 1.04, ß = 0.91 kg 95%CI: 0.25, 1.56 per doubling of PFOA) [29], however body composition by BIA was not assessed [73]. In Project VIVA, participants who became pregnant during postpartum follow up (*n* = 96 pregnancies at 1 year postpartum) were included in analysis if their last delivery was more than 6 months prior. More recently, late pregnancy PFAS (~ 28 weeks), individually and as mixtures were positively associated with postpartum weight retention in the New Hampshire Birth Cohort Study, which also included participants with intervening pregnancies as long as they were not pregnant at postpartum cardiometabolic health survey (31.5%) [33]. In both Project VIVA and NHBCS inclusion of inclusion of women with intervening pregnancies could add error to the outcomes of interest. In contrast, UPSIDE MOMS participants who became pregnant during follow-up were dropped from analysis. Neither previous study examines body composition in the postpartum period, though body fat percentage may more accurately reflect loss of fat mass than weight retention given the contribution of mammary tissue and fluid to weight [69]. Concordance of PPWR and body fat percentage models in the UPSIDE study (in both multivariable linear models and WQS) increases the tenability of our results.

From studies in non-pregnant individuals, there is evidence that PFAS may affect weight gain throughout the lifespan, however mechanisms are not clear. In adolescents, gestational and childhood PFAS exposure has been linked to increased risk of overweight/obesity [16, 17]. In midlife PFAS are associated with weight-related changes in adipokines, and greater risk of cardiovascular disease (CVD, with sex-based differences in some studies) [35, 74–76]. A smaller literature has shown associations in the opposite direction [77]. The biological plausibility of weight modulation by PFAS has been explored in model systems. Though some in vitro work indicates PFAS exposure leads to adipocyte proliferation, [78] there is also evidence that PFAS may increase lipolysis which is a plausible mechanism for exposure induced fat loss [34]. PFAS is known to bind to PPAR-α/γ, and once activated, these markers may lead to hepatic fatty acid oxidation [79]. Another potential route of PFAS-weight modulation is that PFAS have been linked to glucose dysregulation, including gestational diabetes, [61, 73, 80, 81] further demonstrating their metabolism disrupting properties. Additionally, studies in non-pregnant adults have demonstrated associations between PFAS, glucose dysregulation, and metabolic disease [34, 62, 82, 83]. Finally, metabolism may be disrupted through PFAS’ impacts on sex steroid hormones critical to body composition and which change dramatically across pregnancy and the postpartum [43].

Our study has several notable strengths. First, we followed mothers across pregnancy and the postpartum to characterize weight across this critical period. Our evaluation of GWG based on serial clinical measurements was rigorous and included statistical adjustment for missing data, allowing us to accurately quantify total GWG as well as trimester-specific weight gain. Associations between PFAS and reduced GWG were strongest in the third trimester (for example, among BMI < 25 kg/m^2^: PFHxS ß (95% CI) -1.43 kg (-2.82, -0.04) and among BMI >  = 25 kg/m^2^ PFOA ß (95% CI) -1.78 kg (-4.43, 0.87)). Notably, the third trimester is a critical period for fetal growth and alterations during that period may have long-lasting effects on child health [84]. We additionally went beyond standard weight measures used in prior work to assess body composition at two postpartum timepoints. Our well-characterized cohort allowed for the inclusion of several important covariates that have been omitted from prior work, most notably energy intake and physical activity. Given that diet is a primary source of PFAS exposure as well as an important contributor to GWG and PPWR it may be an important source of unmeasured confounding in prior work [39, 40, 85, 86]. Additionally, UPSIDE MOMs had a high rate of breastfeeding in the first year (70% at 6 months, 58% at 12 months). PFAS may be excreted through breastfeeding and contribute to weight loss, by including this covariate we were able to more accurately model associations between PFAS and postpartum outcomes. In general, the results of our mixture analysis corroborated our findings with multivariable models and individual PFAS.

A key limitation of our study is that we measured PFAS at a single timepoint in the second trimester. PFAS are persistent in the body with half-lives estimated to be several years (PFOS: 5.4 years 95%CI; 3.9–6.9, PFOA: 3.8 years 95%CI, 3.1–4.4), [87] and to date, most studies on this topic have assumed consistent concentrations across pregnancy. However recent work examining longitudinal PFAS concentrations across the perinatal period suggests that concentrations may change as blood volume normatively increases and glomerular filtration rate changes in pregnancy [88]. As a result, PFAS concentrations might appear to be lower in participants who gained more weight (and hence experience a greater change in blood volume) in pregnancy, indicating reverse causation. While this is possible, it would likely have minimal impact in our study given that more GWG occurs in the second half of pregnancy after our exposure assessment and indeed, we observed much stronger associations with second and third trimester weight gain.

Another limitation of our study is the use of early pregnancy weight as a proxy for pre-pregnancy weight. While that is a widely accepted practice in pregnancy cohorts, it could lead to BMI group misclassification. Given that earliest pregnancy weight is likely to be a slight overestimate of true pre-pregnancy weight, using it as a proxy for pre-pregnancy weight could artificially reduce PPWR; however, that measurement error is not likely to be associated with PFAS concentrations. In our study, we report -0.02 ± 6.47 kgs retained at 1 year, which may be related to the relatively high pre-pregnancy BMI in our cohort. Other studies have indicated 75% of women are heavier than their pre-pregnancy weight at 1 year postpartum [24]. Given that our study found very little weight retention at 12 months, this potentially limits the generalizability of this cohort. Additionally, our study had loss to follow up. As in many pregnancy cohorts, participants who were older, had more education, and lower BMI were more likely to contribute postpartum data, potentially biasing our results. Given the small number of postnatal participants and the attrition, postpartum results should be interpreted with caution. However we note that directions of association were largely consistent across both the prenatal and postpartum periods which may somewhat ameliorate that concern. Finally, our analysis included a large number of comparisons. To avoid over reliance on p-values, we did not adjust for multiple comparisons instead examining patterns of results (e.g. PFOA and PFHxS were inversely associated with weight gain, weight retention, and body fat) [89].

## Conclusions

In summary, results of this analysis suggest that select PFAS concentrations during gestation are associated with lower GWG and PPWR. Reductions in GWG resulting from PFAS and other environmental exposures are of concern given and may contribute to maternal nutritional deficiency [90]. Additional studies are needed to examine serial PFAS concentrations across pregnancy and the postpartum in relation to GWG, PPWR, and maternal cardiometabolic health in the years following pregnancy. The mechanism of PFAS impact on GWG and PPWR requires further investigation in light of the larger literature suggesting that PFAS may exhibit both obesogenic and anti-obesogenic effects.

### Supplementary Information

**Additional file 1:** **Supplementary Table 1. **Summary of published epidemiological literature on prenatal PFAS concentrations and gestational weight gain and post-partum weight retention. **Supplementary Figure 1. **Histogram of number of clinically recorded weights per participant and table of measurement timing of weights used for weight interpolation. **Supplementary Figure 2. **Directed acyclic graph of covariates considered in the pre- and postnatal analysis. **Supplementary Table 2. **Descriptive Statistics of UPSIDE participants included in the current analysis and parent cohort. **Supplementary Table 3. **Spearman Correlation between log-transformed PFAS (ng/ml) in the UPSIDE cohort. **Supplementary Table 4.** Trimester specific – and total gestational weight gain by body mass index in the UPSIDE cohort. **Supplementary Table 5.** Multivariable linear models examining log-transformed PFAS (ng/ml) in relation to total and trimester-specific gestational weight gain (kg) in the overall UPSIDE cohort and stratified by lower (BMI < 25 kg/m2) versus higher (BMI>=25 kg/m2) early pregnancy BMI. **Supplementary Table 6. **Multivariable linear models examining per interquartile range increase in log-transformed PFAS (ng/ml) in relation to mid/late gestational weight gain (kilograms) in the overall UPSIDE cohort and stratified by lower (BMI < 25 kg/m^2^) versus higher (BMI>=25 kg/m^2^) early pregnancy BMI. **Supplementary Table 7. **Multivariable linear models examining second trimester log-transformed PFAS (ng/ml) in relation to total and trimester-specific gestational weight gain (kg) in the overall UPSIDE cohort and stratified by lower (BMI < 25 kg/m2) versus higher (BMI>=25 kg/m2) early pregnancy BMI, excluding women who went on to deliver preterm (<37 weeks gestation; *n*=13). **Supplementary Table 8. ***p*-value for interaction term (PFAS*pre-pregnancy BMI) in multivariable linear regression models examining log-transformed PFAS (ng/ml) in relation to total gestational weight gain (GWG; in kg), post-partum weight retention (PPWR; in kg) and body fat percentage at 6 and 12 months in the UPSIDE cohort. **Supplementary Table 9. **Spearman-rank correlation between log transformed PFAS (ng/ml) and post-partum weight retention (PPWR, in kg) and body fat percentage in the UPSIDE cohort. **Supplementary Table 10.** Unadjusted and adjusted linear models examining second trimester log-transformed PFAS (ng/ml) in relation to post-partum weight retention (PPWR) in kg and total body fat percentage at 6 and 12 months post-delivery in the UPSIDE cohort. **Supplementary Table 11. **Multivariable linear models examining per interquartile range increase in log-transformed PFAS (ng/ml) in relation to  post-partum weight retention (PPWR) in kg and total body fat percentage at 6 and 12 months post-delivery in the UPSIDE cohort in the overall UPSIDE cohort and stratified by lower (BMI < 25 kg/m^2^) versus higher (BMI>=25 kg/m^2^) early pregnancy BMI^†^. **Supplementary Table 12. **Association between weighted quantile sum of PFAS exposure and prenatal and postpartum maternal weight outcomes in UPSIDE/UPSIDE-Moms cohort. **Supplementary Table 13. **Index weights for individual PFAS in WQS models examining the association between a PFAS mixture and average rate of gain (kg/week) in mid/late pregnancy.

## Data Availability

The raw data from UPSIDE and UPSIDE MOMSs is available from the corresponding author on reasonable request.

## Abbreviations

BIA: Bioelectric impedance analysis
BMI: Body mass index
GWG: Gestational weight gain
SPE: Hybrid solid-phase extraction
IOM: Institute of Medicine
IRBs: Institutional review boards
kcal: Kilocalories
LOD: Limit of detection
METs: Metabolic equivalents of task
NETFOSAA: N-ethyl perfluorooctane sulfonamido acetic acid
NMFOSAA: N-methyl perfluorooctane sulfonamido acetic acid
PFAS: Per- and polyfluoroalkyl substances
PFBS: Perfluorobutanesulfonic acid
PFDA: Perfluorodecanoic acid
PFDODA: Perfluorododecanoic acid
PFHpA: Perfluoroheptanoic acid
PFHxA: Perfluorohexanoic acid
PFHxS: Perfluorohexanesulfonic acid
PFOSA: Perfluorooctanesulfonamide
PFPeA: Perfluoro-n-pentanoic acid
PFOS: Perfluorooctanesulfonic acid
PFOA: Perfluorooctanoic acid
PFNA: Perfluorononanoic acid
PFUNDA: Perfluroundecanoic acid
PPWR: Postpartum weight retention
PPAQ: Pregnancy physical activity questionnaire
UPLC-MS/MS: Ultrahigh-performance liquid chromatography-tandem mass spectrometry
UPSIDE: Understanding Pregnancy Signals and Infant Development
URMC: University of Rochester medical center

## References

[1] Brase RA, Mullin EJ, Spink DC (2021). Legacy and emerging per- and polyfluoroalkyl substances: analytical techniques, environmental fate, and health effects. Int J Mol Sci.

[2] Buck RC, Franklin J, Berger U (2011). Perfluoroalkyl and polyfluoroalkyl substances in the environment: terminology, classification, and origins. Integr Environ Assess Manag.

[3] NIOSH. Per- and polyfluoroalkyl substances (PFAS). https://www.cdc.gov/niosh/topics/pfas/default.html. Published 2023. Updated September 15, 2022. Accessed 23 Feb 2023.

[4] EPA. "EPA and 3M announce phase out of PFOS." Washington DC: US Environmental Protection Agency Press Release; 2000.

[5] Dassuncao C, Hu XC, Nielsen F, Weihe P, Grandjean P, Sunderland EM (2018). Shifting global exposures to poly- and perfluoroalkyl substances (PFASs) evident in longitudinal birth cohorts from a seafood-consuming population. Environ Sci Technol.

[6] Sunderland EM, Hu XC, Dassuncao C, Tokranov AK, Wagner CC, Allen JG (2019). A review of the pathways of human exposure to poly- and perfluoroalkyl substances (PFASs) and present understanding of health effects. J Expo Sci Environ Epidemiol.

[7] Qian Wu KK. Perfluoroalkyl Substances (PFASs) in Foodstuffs and Human Dietary Exposure, Advances in the Determination of Xenobiotics in Foods Current and Future Developments in Food Science. In: Gomara BaM, M.L., ed.: Bentham Science Publisher; 2019. p. 1-259.

[8] Meeker JD (2012). Exposure to environmental endocrine disruptors and child development. Arch Pediatr Adolesc Med.

[9] Barrett ES, Groth SW, Preston EV, Kinkade C, James-Todd T (2021). Endocrine-disrupting chemical exposures in pregnancy: a sensitive window for later-life cardiometabolic health in women. Curr Epidemiol Rep.

[10] Lite C, Raja GL, Juliet M (2022). In utero exposure to endocrine-disrupting chemicals, maternal factors and alterations in the epigenetic landscape underlying later-life health effects. Environ Toxicol Pharmacol.

[11] Zhuang LH, Chen A, Braun JM (2021). Effects of gestational exposures to chemical mixtures on birth weight using Bayesian factor analysis in the Health Outcome and Measures of Environment (HOME) Study. Environ Epidemiol.

[12] Yao Q, Gao Y, Zhang Y, Qin K, Liew Z, Tian Y (2021). Associations of paternal and maternal per- and polyfluoroalkyl substances exposure with cord serum reproductive hormones, placental steroidogenic enzyme and birth weight. Chemosphere.

[13] Fan X, Tang S, Wang Y (2022). Global exposure to per- and polyfluoroalkyl substances and associated burden of low birthweight. Environ Sci Technol.

[14] Chang CJ, Barr DB, Ryan PB (2022). Per- and polyfluoroalkyl substance (PFAS) exposure, maternal metabolomic perturbation, and fetal growth in African American women: a meet-in-the-middle approach. Environ Int.

[15] Gardener H, Sun Q, Grandjean P (2021). PFAS concentration during pregnancy in relation to cardiometabolic health and birth outcomes. Environ Res.

[16] Geiger SD, Yao P, Vaughn MG, Qian Z (2021). PFAS exposure and overweight/obesity among children in a nationally representative sample. Chemosphere.

[17] Braun JM, Eliot M, Papandonatos GD (2021). Gestational perfluoroalkyl substance exposure and body mass index trajectories over the first 12 years of life. Int J Obes (Lond).

[18] Groth SW, Fernandez ID, Block RC (2021). Biological changes in the pregnancy-postpartum period and subsequent cardiometabolic risk-UPSIDE MOMS: a research protocol. Res Nurs Health.

[19] Stuart JJ, Tanz LJ, Rimm EB (2022). Cardiovascular risk factors mediate the long-term maternal risk associated with hypertensive disorders of pregnancy. J Am Coll Cardiol.

[20] Tanz LJ, Stuart JJ, Williams PL (2017). Preterm delivery and maternal cardiovascular disease in young and middle-aged adult women. Circulation.

[21] Rasmussen KM, Yaktine AL, IOM (2009). The National academies collection: reports funded by National Institutes of Health. Weight gain during pregnancy: reexamining the guidelines.

[22] Goldstein RF, Abell SK, Ranasinha S (2017). Association of gestational weight gain with maternal and infant outcomes: a systematic review and meta-analysis. JAMA.

[23] Gore SA, Brown DM, West DS (2003). The role of postpartum weight retention in obesity among women: a review of the evidence. Ann Behav Med.

[24] Endres LK, Straub H, McKinney C (2015). Postpartum weight retention risk factors and relationship to obesity at 1 year. Obstet Gynecol.

[25] Hutchins F, Abrams B, Brooks M (2020). The effect of gestational weight gain across reproductive history on maternal body mass index in midlife: the study of women's health across the nation. J Womens Health (Larchmt).

[26] Haugen M, Brantsæter AL, Winkvist A (2014). Associations of pre-pregnancy body mass index and gestational weight gain with pregnancy outcome and postpartum weight retention: a prospective observational cohort study. BMC Pregnancy Childbirth.

[27] Bitan R, Miodownik S, Pariente G (2022). Gestational weight gain and long-term postpartum weight retention. Harefuah.

[28] Ashley-Martin J, Dodds L, Arbuckle TE (2016). Maternal and neonatal levels of perfluoroalkyl substances in relation to gestational weight gain. Int J Environ Res Public Health.

[29] Mitro SD, Sagiv SK, Rifas-Shiman SL (2020). Per- and polyfluoroalkyl substance exposure, gestational weight gain, and postpartum weight changes in project viva. Obesity (Silver Spring).

[30] Romano ME, Gallagher LG, Eliot MN (2021). Per- and polyfluoroalkyl substance mixtures and gestational weight gain among mothers in the Health Outcomes and Measures of the Environment study. Int J Hyg Environ Health.

[31] Marks KJ, Jeddy Z, Flanders WD (2019). Maternal serum concentrations of perfluoroalkyl substances during pregnancy and gestational weight gain: the avon longitudinal study of parents and children. Reprod Toxicol.

[32] Jaacks LM, Boyd Barr D, Sundaram R, Grewal J, Zhang C, Buck Louis GM (2016). Pre-pregnancy maternal exposure to persistent organic pollutants and gestational weight gain: a prospective cohort study. Int J Environ Res Public Health.

[33] Wang Y, Howe C, Gallagher LG, et al. Per- and Polyfluoroalkyl Substances (PFAS) mixture during pregnancy and postpartum weight retention in the New Hampshire Birth Cohort Study (NHBCS). Toxics*.* 2023;11(5). 10.3390/toxics11050450. 10.3390/toxics11050450 PMC10223499 37235264

[34] Chen Z, Yang T, Walker DI (2020). Dysregulated lipid and fatty acid metabolism link perfluoroalkyl substances exposure and impaired glucose metabolism in young adults. Environ Int.

[35] Ding N, Karvonen-Gutierrez CA, Herman WH, Calafat AM, Mukherjee B, Park SK (2021). Perfluoroalkyl and polyfluoroalkyl substances and body size and composition trajectories in midlife women: the study of women’s health across the nation 1999–2018. Int J Obes.

[36] Domazet SL, Jensen TK, Wedderkopp N, Nielsen F, Andersen LB, Grøntved A (2020). Exposure to perfluoroalkylated substances (PFAS) in relation to fitness, physical activity, and adipokine levels in childhood: The european youth heart study. Environ Res.

[37] Fassler CS, Pinney SE, Xie C, Biro FM, Pinney SM (2019). Complex relationships between perfluorooctanoate, body mass index, insulin resistance and serum lipids in young girls. Environ Res.

[38] Pinney SM, Windham GC, Xie C (2019). Perfluorooctanoate and changes in anthropometric parameters with age in young girls in the Greater Cincinnati and San Francisco Bay Area. Int J Hyg Environ Health.

[39] Eick SM, Goin DE, Trowbridge J, et al. Dietary predictors of prenatal per- and poly-fluoroalkyl substances exposure. J Expo Sci Environ Epidemiol. 2023;33:32–9. 10.1038/s41370-021-00386-6. 10.1038/s41370-021-00386-6 PMC8983786 34615969

[40] Stuebe AM, Oken E, Gillman MW (2009). Associations of diet and physical activity during pregnancy with risk for excessive gestational weight gain. Am J Obstet Gynecol.

[41] Gardner B, Wardle J, Poston L, Croker H (2011). Changing diet and physical activity to reduce gestational weight gain: a meta-analysis. Obes Rev.

[42] O’Connor T, Best M, Brunner J (2021). Cohort profile: Understanding Pregnancy Signals and Infant Development (UPSIDE): a pregnancy cohort study on prenatal exposure mechanisms for child health. BMJ Open.

[43] Rivera-Núñez Z, Kinkade CW, Khoury L (2023). Prenatal perfluoroalkyl substances exposure and maternal sex steroid hormones across pregnancy. Environ Res.

[44] Honda M, Robinson M, Kannan K (2018). A rapid method for the analysis of perfluorinated alkyl substances in serum by hybrid solid-phase extraction. Environ Chem.

[45] Krukowski RA, West DS, DiCarlo M (2016). Are early first trimester weights valid proxies for preconception weight?. BMC Pregnancy Childbirth.

[46] Rangel BousquetCarrilho T, M, Rasmussen K, Rodrigues Farias D (2020). Agreement between self-reported pre-pregnancy weight and measured first-trimester weight in Brazilian women. BMC Pregnancy and Childbirth.

[47] Inskip H, Crozier S, Baird J (2021). Measured weight in early pregnancy is a valid method for estimating pre-pregnancy weight. J Dev Orig Health Dis.

[48] Farbo DJ, Rhea DJ (2021). A pilot study examining body composition classification differences between body mass index and bioelectrical impedance analysis in children with high levels of physical activity. Front Pediatrics.

[49] Bertoli S, Leone A, Vignati L (2016). Metabolic correlates of subcutaneous and visceral abdominal fat measured by ultrasonography: a comparison with waist circumference. Nutr J.

[50] Shiao S-YPK, Andrews CM, Helmreich RJ (2005). Maternal race/ethnicity and predictors of pregnancy and infant outcomes. Biol Res Nurs.

[51] Burris HH, Collins JW, Wright RO (2011). Racial/ethnic disparities in preterm birth: clues from environmental exposures. Curr Opin Pediatr.

[52] Wang E, Glazer KB, Howell EA, Janevic TM (2020). Social determinants of pregnancy-related mortality and morbidity in the united states: a systematic review. Obstet Gynecol.

[53] Chasan-Taber L, Schmidt MD, Roberts DE, Hosmer D, Markenson G, Freedson PS (2004). Development and validation of a pregnancy physical activity questionnaire. Med Sci Sports Exerc.

[54] Tooze JA, Midthune D, Dodd KW (2006). A new statistical method for estimating the usual intake of episodically consumed foods with application to their distribution. J Am Diet Assoc.

[55] Feskanich D, Sielaff BH, Chong K, Buzzard IM (1989). Computerized collection and analysis of dietary intake information. Comput Methods Programs Biomed.

[56] Carrico C, Gennings C, Wheeler DC, Factor-Litvak P (2015). Characterization of weighted quantile sum regression for highly correlated data in a risk analysis setting. J Agric Biol Environ Stat.

[57] Johnson PI, Sutton P, Atchley DS (2014). The Navigation Guide - evidence-based medicine meets environmental health: systematic review of human evidence for PFOA effects on fetal growth. Environ Health Perspect.

[58] Wikström S, Lin P-I, Lindh CH, Shu H, Bornehag C-G (2020). Maternal serum levels of perfluoroalkyl substances in early pregnancy and offspring birth weight. Pediatr Res.

[59] Koustas E, Lam J, Sutton P (2014). The Navigation Guide - evidence-based medicine meets environmental health: systematic review of nonhuman evidence for PFOA effects on fetal growth. Environ Health Perspect.

[60] Blake BE, Fenton SE (2020). Early life exposure to per- and polyfluoroalkyl substances (PFAS) and latent health outcomes: a review including the placenta as a target tissue and possible driver of peri- and postnatal effects. Toxicology.

[61] Ashley-Martin J, Dodds L, Arbuckle TE (2017). Maternal concentrations of perfluoroalkyl substances and fetal markers of metabolic function and birth weight. Am J Epidemiol.

[62] Lin PD, Cardenas A, Hauser R (2021). Temporal trends of concentrations of per- and polyfluoroalkyl substances among adults with overweight and obesity in the United States: results from the Diabetes Prevention Program and NHANES. Environ Int.

[63] NHANES. National Report on Human Exposure to Environmental Chemicals. https://www.cdc.gov/exposurereport/data_tables.html. Accessed 20 Jan 2023.

[64] Gore AC, Chappell VA, Fenton SE (2015). EDC-2: the endocrine society's second scientific statement on endocrine-disrupting chemicals. Endocr Rev.

[65] Lee YJ, Jung HW, Kim HY, Choi YJ, Lee YA (2021). Early-life exposure to per- and poly-fluorinated alkyl substances and growth, adiposity, and puberty in children: a systematic review. Front Endocrinol (Lausanne).

[66] Park SK, Ding N, Han D (2021). Perfluoroalkyl substances and cognitive function in older adults: Should we consider non-monotonic dose-responses and chronic kidney disease?. Environ Res.

[67] S. K (2017). Effect of diet and physical activity based interventions in pregnancy on gestational weight gain and pregnancy outcomes: meta-analysis of individual participant data from randomised trials. BMJ.

[68] Skouteris H, Hartley-Clark L, McCabe M (2010). Preventing excessive gestational weight gain: a systematic review of interventions. Obes Rev.

[69] Gilmore LA, Klempel-Donchenko M, Redman LM (2015). Pregnancy as a window to future health: excessive gestational weight gain and obesity. Semin Perinatol.

[70] Etchison WC, Bloodgood EA, Minton CP (2011). Body mass index and percentage of body fat as indicators for obesity in an adolescent athletic population. Sports Health.

[71] Si S, Tewara MA, Ji X (2020). Body surface area, height, and body fat percentage as more sensitive risk factors of cancer and cardiovascular disease. Cancer Med.

[72] Deurenberg-Yap M, Chew SK, Deurenberg P (2002). Elevated body fat percentage and cardiovascular risks at low body mass index levels among Singaporean Chinese Malays Indians. Obes Rev.

[73] Mitro SD, Sagiv SK, Fleisch AF (2020). Pregnancy per- and polyfluoroalkyl substance concentrations and postpartum health in project viva: a prospective cohort. J Clin Endocrinol Metab.

[74] Ding N, Karvonen-Gutierrez CA, Herman WH, Calafat AM, Mukherjee B, Park SK (2021). Associations of perfluoroalkyl and polyfluoroalkyl substances (PFAS) and PFAS mixtures with adipokines in midlife women. Int J Hyg Environ Health.

[75] Shankar A, Xiao J, Ducatman A (2012). Perfluorooctanoic acid and cardiovascular disease in US adults. Arch Intern Med.

[76] Feng X, Long G, Zeng G, Zhang Q, Song B, Wu K-H (2022). Association of increased risk of cardiovascular diseases with higher levels of perfluoroalkylated substances in the serum of adults. Environ Sci Pollut Res.

[77] Honda-Kohmo K, Hutcheson R, Innes KE, Conway BN (2019). Perfluoroalkyl substances are inversely associated with coronary heart disease in adults with diabetes. J Diabetes Complications.

[78] Watkins AM, Wood CR, Lin MT, Abbott BD (2015). The effects of perfluorinated chemicals on adipocyte differentiation in vitro. Mol Cell Endocrinol.

[79] Patsouris D, Mandard S, Voshol PJ (2004). PPARalpha governs glycerol metabolism. J Clin Invest.

[80] Birru RL, Liang HW, Farooq F (2021). A pathway level analysis of PFAS exposure and risk of gestational diabetes mellitus. Environ Health.

[81] Matilla-Santander N, Valvi D, Lopez-Espinosa MJ (2017). Exposure to perfluoroalkyl substances and metabolic outcomes in pregnant women: evidence from the Spanish INMA birth cohorts. Environ Health Perspect.

[82] Yu S, Feng WR, Liang ZM (2021). Perfluorooctane sulfonate alternatives and metabolic syndrome in adults: new evidence from the Isomers of C8 Health Project in China. Environ Pollut.

[83] Zhang YT, Zeeshan M, Su F (2022). Associations between both legacy and alternative per- and polyfluoroalkyl substances and glucose-homeostasis: the Isomers of C8 health project in China. Environ Int.

[84] Silva CCV, El Marroun H, Sammallahti S (2021). Patterns of fetal and infant growth and brain morphology at age 10 years. JAMA Netw Open.

[85] Domingo JL, Nadal M (2017). Per- and Polyfluoroalkyl Substances (PFASs) in food and human dietary intake: a review of the recent scientific literature. J Agric Food Chem.

[86] Rolfo A, Nuzzo AM, De Amicis R, Moretti L, Bertoli S, Leone A (2020). Fetal-maternal exposure to endocrine disruptors: correlation with diet intake and pregnancy outcomes. Nutrients.

[87] Olsen GW, Burris JM, Ehresman DJ (2007). Half-life of serum elimination of perfluorooctanesulfonate, perfluorohexanesulfonate, and perfluorooctanoate in retired fluorochemical production workers. Environ Health Perspect.

[88] Oh J, Bennett DH, Tancredi DJ (2022). Longitudinal changes in maternal serum concentrations of per- and polyfluoroalkyl substances from pregnancy to two years postpartum. Environ Sci Technol.

[89] Rothman KJ (1990). No adjustments are needed for multiple comparisons. Epidemiology.

[90] Campos CAS, Malta MB, Neves PAR, Lourenço BH, Castro MC, Cardoso MA (2019). Gestational weight gain, nutritional status and blood pressure in pregnant women. Rev Saúde Pública.

