# Supplementary material for: Per- and polyfluoroalkyl substances, gestational weight gain, postpartum weight retention and body composition in the UPSIDE cohort
Source: Environ Health. 2023 Sep 2;22:61. doi: 10.1186/s12940-023-01009-3 (PMC10474772; doi:10.1186/s12940-023-01009-3)

Supplementary Table 1. Summary of published epidemiological literature on prenatal PFAS concentrations and gestational weight gain and post-partum weight retention.

Cohort, Author (Year)	N	Sampling Period	Gestational Age at Sampling Time	Cohort BMI Profile	Median PFAS Concentrations (ng/ml)	Beta for GWG (95% CI)						Adjusted for:
						Unit	PFOS	PFOA	PFNA	PFHXS	EtFOSAA	
**Gestational Weight Gain**												
MIREC, Ashley-Martin (2016)	1036	2008-2011	1st trimester	Normal: 61%, Overweight: 22%, Obese: 14%	PFOA: 1.7 PFOS: 4.6 PFHxS: 1.0	kg (log2)	**BMI<25: 0.39 (0.02, 0.75)**	BMI<25: 0.38 (-0.03, 0.79)		BMI<25: 0.12 (-0.14, 0.38)		Age, income, parity
							BMI>=25: -0.08 (-0.94, 0.78)	BMI>=25: 0.58 (-0.26, 1.42)		BMI>=25: -0.12 (-0.73, 0.48)		
LIFE, Jaacks (2016)	258	2005-2007	pre-pregnancy	IOM Adequate mean BMI: 24.2, IOM inadequate mean BMI 26: IOM excessive mean BMI: 28	Mean PFOA: 3.7 PFOS: 14.8 PFNA: 1.4 EtFOSAA: 0.03	lbs (natural log)	0.26 (-0.66, 1.18)	0.09 (-0.84, 1.02)	-0.03 (-1.00, 0.94)			BMI, serum lipids
AVON, Marks (2020)	14,451	1991-1992	Median: 18, IQR 11,32 weeks	Underweight: 11%, Normal: 68%, Overweight: 15%, Obese: 5.3%	PFOA: 3.0 PFOS: 13.8 PFHxS: 1.9 PFNA: 0.4	kg (natural log)	BMI<25: 0.01 (-0.08, 0.09)	BMI<25: 0.01 (-0.08, 0.09)	**BMI<25: 0.09 (0.02, 0.16)**	BMI<25: 0.00 (-0.05, 0.05)		Education, smoking, age, parity, BMI, gestational age, sample collection
							BMI>=:-0.12 (-0.30, 0.06)	BMI>=: -0.20 (-0.41, 0.02)	BMI>=25: -0.12 (-0.31, 0.07)	BMI>=: 0.02 (-0.08, 0.11)		
VIVA, Mitro (2020)	1,614	1999-2002	Median 9.7, Range 4.8-21.4 weeks	Normal: 62%, Overweight: 22 %, Obese: 16%	PFOA: 5.9 PFOS: 25.7 PFHxS: 2.5 EtFOSAA: 1.2	Kg (log2)	0.01 (-0.34, 0.36)	0.26 (-0.15, 0.66)	0.10 (-0.26, 0.45)	0.01 (-0.25, 0.27)	**0.37 (0.11, 0.62)**	Age, BMI, marital status, race ethnicity, education, income, smoking, parity
HOME, Romano (2021)	150	2003-2006	18 ± 5 weeks	Normal: 54%, Overweight: 27%, Obese 19%	PFOA: 5.4 PFOS: 13.7 PFHxS: 1.5 PFNA: 0.9	lbs (log2),	BMI<25: 0.48 (-1.31, 2.27)	BMI<25: 0.59 (-1.03, 2.21)	BMI<25: -0.99 (-3.80, 1.82)	BMI<25: -0.54 (-1.93, 0.84)		Education, race, BMI, income, cotinine, alcohol, sample collection, parity, gestational age at delivery
							BMI>=25: 0.64 (-1.18, 2.45)	BMI>=25: 1.09 (-0.61, 2.79)	BMI>=25: 2.63 (-0.75, 6.00)	BMI>=25: 0.48 (-1.23, 2.19)		
**Postpartum Weight Retention**												
NHBCS, Wang (2023)	482	2009-2018	Mean 28 weeks	Mean BMI 26.6 kg/m^2	PFOA: 1.1 PFOS: 3.2 PFHxS: 0.7 PFNA: 0.5	kg (log2), beta per doubling	**1.76 (0.31, 3.22)**	1.39 (−0.27, 3.04)		1.04 (−0.19, 2.28)		Age, education, race/ethnicity, marital status, BMI, smoking, lactation, healthy eating index, physical activity, week of blood sampling, enrollment year, additional pregnancies
VIVA, Mitro (2020)	860	1999-2002	Median 9.7; Range 4.8-21.4 weeks	Normal: 62%, Overweight: 22 %, Obese: 16%	PFOA: 3.0 PFOS: 13.8 PFHxS: 1.9 PFNA: 0.4	Kg (log2)	1Y: 0.22 (-0.20, 0.64)	**1Y: 0.55 (0.07, 1.04)**	1Y: 0.00 (-0.44, 0.44)	1Y: 0.18 (-0.11, 0.48)	1Y: 0.13 (-0.19, 0.44)	Age, BMI, marital status, race ethnicity, education, income, smoking, parity
							**3Y: 0.63 (0.04, 1.22)**	**3Y: 0.91 (0.25, 1.56)**	3Y: 0.09 (-0.49, 0.66)	3Y: 0.17 (-0.27, 0.60)	3Y: 0.40 (-0.01, 0.82)	

Abbr: BMI: body mass index, IOM: Institute of Medicine, IQR: interquartile range, PFAS: per- and poly-fluoroalkyl substances; PFOA: perfluorooctanoic acid; PFOS: perfluorooctanesulfonic acid; PFNA: perfluorononanoic acid; PFHxS: perfluorohexane sulfonic acid; EtFOSAA: 2-(N-ethyl-perfluorooctane sulfonamido) acetic acid, Y: years. Bold denotes significance at $p<0.05$.

Supplementary Table 2. **Descriptive Statistics of UPSIDE participants included in the current analysis and parent cohort.**

	Group 1. Participants contributing prenatal data (n=243)	Group 2. Participants contributing only prenatal data (n=109)	Group 3. Participants contributing prenatal and postpartum data (n=139)†	p-value (Group 2 and 3)
**Demographic and lifestyle measures- continuous**	**Mean (SD)**			
Age (years)	29.3 (4.4)	28.4 (4.6)	30.0 (4.2)	**0.005**
Pre-pregnancy BMI (kg/m^2)	27.9 (7.2)	29.2 (7.7)	27.1 (6.8)	**0.026**
Gestational age at delivery (wks)	39.5 (1.4)	39.2 (1.6)	39.7 (1.2)	**0.006**
Gestational age at PFAS measurement (wks)	21.1 (1.8)	21.3 (2.0)	21.1 (1.6)	0.268
Prenatal Energy Intake (kcal/day)	2157.6 (311.8)	2153.2 (327.4)	2173.5 (323.3)	0.628
Postpartum Energy Intake (kcal/day)	-	-	1946.1 (408.6)	-
Prenatal Physical Activity (METs/day)	341.5 (173.9)	354.9 (186.0)	338.8 (171.2)	0.485
Postpartum Physical Activity (METs/week) – 6 months	-	-	371.4 (158.2)	-
Postpartum Physical Activity (METs/week) – 12 months	-	-	391.5 (199.0)	-
Infant birthweight (grams)	3386.6 (520.2)	3290.3 (567.4)	3464.4.6 (474.3.7)	**0.011**
**Demographic and lifestyle measures- categorical**	**n (%)**			
Preterm birth	13 (5.3)	12 (11.0)	0	
Race/Ethnicity				
Non-Hispanic White	151 (62.1)	56 (51.4)	96 (69.1)	**0.009**
Non-Hispanic Black	51 (21.0)	28 (25.7)	27 (19.4)	0.078
Other	41 (16.9)	25 (22.9)	16 (11.5)	**0.016**
Parity				
Nulliparous	81 (33.3)	38 (34.9)	45 (32.4)	0.513
Parous	162 (66.7)	71 (65.1)	94 (67.6)	0.949
Education				
HS or less	78 (32.1)	46 (42.2)	34 (24.5)	**0.001**
More than HS	165 (67.9)	63 (57.8)	105 (75.5)	**0.014**
Smoking during Pregnancy (any)	15 (6.2)	9 (8.3)	7 (5.0)	0.196
Infant sex (male)	124 (51.0)	52 (47.7)	74 (53.2)	0.598
Breastfeeding (any) – 6 months	-	-	87 (70.1)	-
Breastfeeding (any) –12 months	-	-	60 (57.7)	-
**Gestational weight gain measures (in kg)**	**Mean (SD)**			
1st Trimester	0.69 (2.29)	0.73 (2.19)	0.66 (2.35)	0.815
Average weekly – 1st Trimester	0.13 (0.34)	0.11 (0.31)	0.13 (0.35)	0.690
2nd Trimester	6.26 (3.22)	5.81 (3.41)	6.51 (3.06)	0.095
Average weekly – 2nd Trimester	0.45 (0.23)	0.41 (0.24)	0.47 (0.22)	0.090
3rd Trimester	5.27 (3.06)	4.95 (3.22)	5.47 (2.95)	0.192
Average weekly – 3rd Trimester	0.48 (0.28)	0.47 (0.31)	0.49 (0.25)	0.585
Mid/Late	11.53 (5.29)	10.76 (5.64)	12.06 (4.96)	0.060
Average weekly – Mid/Late	0.46 (0.21)	0.44 (0.23)	0.48 (0.19)	0.133
Total	12.22 (6.27)	11.49 (6.48)	12.73 (6.05)	0.124
Average weekly – across pregnancy	0.39 (0.20)	0.37 (0.21)	0.40 (0.19)	0.174
**GWG by IOM recommendations**	**n (%)**			
Less than IOM recommended	50 (20.6)	25 (22.9)	24 (18.0)	0.266
IOM recommended	97 (39.9)	41 (37.6)	56 (42.1)	0.669
Greater than IOM recommended	96 (39.5)	43 (39.4)	53 (39.8)	0.832
**Postpartum weight retention and adiposity**	**Mean (SD)**			
Weeks since delivery – 6 months	-	-	27.6 (3.8)	-
Weeks since delivery – 12 months	-	-	55.4 (5.6)	-
Weight retention (kg) – 6 months	-	-	0.98 (5.35)	-
Weight retention (kg) – 12 months	-	-	-0.02 (6.47)	-
Body fat (%) – 6 months	-	-	31.9 (8.3)	-
Body fat (%) – 12 months	-	-	31.8 (8.9)	-
PFAS concentrations	**Geometric Mean (GSD)**			
PFOS	2.50 (1.63)	2.50 (1.60)	2.49 (1.67)	0.634
PFOA	0.57 (1.86)	0.58 (1.83)	0.57 (1.93)	0.990
PFNA	0.26 (1.73)	0.26 (1.65)	0.26 (1.84)	0.489
PFHxS	1.74 (1.52)	1.67 (1.56)	1.79 (1.49)	0.184
PFDA	0.05 (2.33)	0.05 (2.20)	0.05 (2.57)	0.559

Abbreviations: BMI: body mass index; GWG: gestational weight gain, GSD: geometric standard deviation, HS: high school, IOM: Institute of Medicine; kcal: kilocalories; kg: kilograms, MET: metabolic equivalent of task, PFAS: per- and poly-fluoroalkyl substances, PFDA: perfluorodecanoic acid; PFHxS: perfluorohexanesulfonic acid, PFNA: perfluorononanoic acid; PFOA: perfluorooctanoic acid; PFOS: perfluorooctanesulfonic acid.

† N=5 participants included in the postnatal analysis were not included in prenatal analysis. The number of participants evaluated at 6 and 12 months postpartum was 124 and 104, respectively. Bold indicates $p<0.05$.

Supplementary Table 3. Spearman Correlation between log-transformed PFAS (ng/ml) in the UPSIDE cohort.

	PFOS	PFOA	PFNA	PFHxS	PFDA
**PFOS**	1.00	0.56***	0.54***	0.43***	0.43***
**PFOA**	0.56***	1.00	0.58***	0.35***	0.32***
**PFNA**	0.54***	0.58***	1.00	0.26***	0.58***
**PFHxS**	0.43***	0.35***	0.26***	1.00	0.15*
**PFDA**	0.42***	0.32***	0.58***	0.15*	1.00

* indicates p<0.05, ** indicates p<0.01, ***indicates p<0.001..

Abbr: PFAS: per- and poly-fluoroalkyl substances, PFDA: perfluorodecanoic acid; PFHxS: perfluorohexanesulfonic acid, PFNA: perfluorononanoic acid; PFOA: perfluorooctanoic acid; PFOS: perfluorooctanesulfonic acid.

Supplementary Table 4. Trimester specific – and total gestational weight gain by body mass index in the UPSIDE cohort.

	Participants contributing prenatal data		Participants contributing postpartum data	
	Pre-pregnancy BMI <25 kg/m^2 (n=114)	Pre-pregnancy BMI >= 25 kg/m^2 (n=129)	Pre-pregnancy BMI <25 kg/m^2 (n=72)	Pre-pregnancy BMI >= 25 kg/m^2 (n=67)
Gestational weight gain measures	Mean (SD)			
1st Trimester (kg)	1.35 (1.81)	0.10 (2.50)	1.41 (1.78)	-0.14 (2.63)
Average weekly – 1st Trimester (kg)	0.24 (0.29)	0.02 (0.35)	0.26 (0.31)	0.00 (0.35)
2nd Trimester (kg)	7.57 (2.38)	5.09 (3.42))	7.45 (2.29)†	5.48 (3.46)‡
Average weekly – 2nd Trimester (kg)	0.54 (0.17)	0.36 (0.24)	0.53 (0.16)	0.39 (0.25)
3rd Trimester (kg)	5.60 (2.42)	4.99 (3.52)	5.68 (2.49)†	5.24 (3.38)*
Average weekly – 3rd Trimester (kg)	0.50 (0.21)	0.46 (0.33)	0.50 (0.20)	0.47 (0.30)
Mid/late (kg)	13.17 (3.78)	10.08 (5.99)	13.22 (3.49)	10.79 (5.95)
Average weekly – Mid/late(kg)	0.52 (0.15)	0.40 (0.24)	0.52 (0.13)	0.43 (0.23)
Total (kg)	14.52 (4.25)	10.18 (7.03)	14.66 (3.93)†	10.63 (7.19)*
Average weekly – across pregnancy	0.46 (0.14)	0.32 (0.22)	0.46 (0.13)	0.33 (0.23)
	n (%)			
Less than IOM recommended	18 (15.8)	32 (24.8)	12 (16.9) †	13 (20.0)*
IOM recommended	62 (54.4)	35 (27.1)	37 (52.1) †	21 (32.3)*
Greater than IOM recommended	34 (29.8)	62 (48.1)	22 (31.0) †	31 (47.7)*

Abbreviations: BMI: body mass index; GWG: gestational weight gain, IOM: Institute of Medicine.
†n=71,‡n=66, *n=65.

Supplementary Table 5. Multivariable linear models examining log-transformed PFAS (ng/ml) in relation to total and trimester-specific gestational weight gain (kilograms) and rate (kg/week) in the overall UPSIDE cohort and stratified by lower (BMI < 25 kg/m^2) versus higher (BMI>=25 kg/m^2) early pregnancy BMI[†,*].

Term	Model	PFOS ß (95% CI)	PFOA ß (95% CI)	PFNA ß (95% CI)	PFHxS ß (95% CI)	PFDA ß (95% CI)
**All Participants (n=243)**						
**Total**	*Unadjusted*	1.11 (-0.50, 2.74)	-0.02 (-1.31, 1.27)	0.35 (-1.11, 1.80)	1.14 (-0.74, 3.02)	**1.05 (0.12, 1.98)**
	Minimally Adjusted	-0.11 (-1.61, 1.40)	**-1.54 (-2.82, -0.27)**	-0.14 (-1.46, 1.18)	-1.65 (-3.48 (0.21)	0.15 (-0.71, 1.01)
	Fully Adjusted	0.10 (-1.37, 1.57)	**-1.54 (-2.79, -0.30)**	-0.26 (-1.55, 1.03)	-1.59 (-3.39, 0.21)	0.21 (-0.62, 1.05)
	Rate (kg/week)	0.00 (-0.05, 0.04)	**-0.05 (-0.09, -0.01)**	-0.01 (-0.05, 0.03)	-0.05 (-0.11, 0.01)	0.01 (-2.06, 0.03)
**Mid/Late**	*Unadjusted*	0.66 (-0.71, 2.03)	-0.13 (-1.22, 0.96)	0.05 (-1.18, 1.28)	0.80 (-0.80, 2.39)	0.68 (-0.11, 1.47)
	Minimally Adjusted	-0.33 (-1.63, 0.96)	**-1.43 (-2.53, -0.33)**	-0.41 (-1.55, 0.73)	-1.40 (-3.00, 0.19)	-0.02 (-0.76, 0.72)
	Fully Adjusted	-0.13 (-1.39, 1.14)	**-1.40 (-2.47, -0.33)**	-0.49 (-1.60, 0.62)	-1.34 (-2.89, 0.21)	0.04 (-0.68, 0.76)
	Rate (kg/week)	-0.00 (-0.05, 0.05)	**-0.05 (-0.10, -0.01)**	-0.02 (-0.06, 0.02)	-0.05 (-1.13, 0.01)	0.00 (-0.03, 0.03)
**3rd Tri**	*Unadjusted*	0.07 (-0.72, 0.86)	-0.12 (-0.74, 0.51)	0.06 (-0.65, 0.77)	-0.20 (-1.12, 0.72)	0.28 (-0.18, 0.73)
	Minimally Adjusted	-0.24 (-1.05, 0.57)	-0.61 (-1.30, 0.08)	-0.18 (-0.89, 0.53)	-0.88 (-1.87, 0.11)	0.13 (-0.33, 0.59)
	Fully Adjusted	-0.20 (-0.99, 0.60)	-0.62 (-1.30, 0.05)	-0.23 (-0.93, 0.47)	-0.90 (-1.87, 0.08)	0.15 (-0.31, 0.60)
	Rate (kg/week)	-0.01 (-0.08, 0.07)	-0.04 (-0.10, 0.02)	-0.02 (-0.08, 0.05)	-0.06 (-0.15, 0.03)	0.02 (-0.03, 0.06)
**2nd Tri**	*Unadjusted*	0.59 (-0.24, 1.42)	-0.01 (-0.68, 0.65)	0.00 (-0.75, 0.75)	**1.00 (0.03, 1.96)**	0.40 (-0.08, 0.88)
	Minimally Adjusted	-0.09 (-0.83, 0.65)	**-0.82 (-1.44, -0.19)**	-0.23 (-0.88, 0.42)	-0.53 (-1.44, 0.38)	-0.15 (-0.56, 0.28)
	Fully Adjusted	-0.02 (-0.74, 0.70)	**-0.84 (-1.54, -0.23)**	-0.31 (-0.94, 0.32)	-0.52 (-1.41, 0.37)	-0.12 (-0.53, 0.30)
	Rate (kg/week)	0.00 (-0.05, 0.05)	**-0.06 (-0.10, -0.02)**	-0.02 (-0.07, 0.02)	-0.04 (-0.10, 0.03)	-0.01 (-0.04, 0.02)
**BMI<25 kg/m (n=114)**						
**Total**	*Unadjusted*	**-1.96 (-3.74, -0.19)**	-1.03 (-2.29, 0.22)	**-2.16 (-3.75, -0.57)**	-1.56 (-3.83, 0.71)	**-1.04 (-2.00, -0.08)**
	Minimally Adjusted	**-2.24 (-4.16, -0.32)**	-1.43 (-2.92, 0.05)	**-2.27 (-4.01, -0.53)**	-2.04 (-4.57, 0.48)	**-1.15 (-2.17, -0.14)**
	Fully Adjusted	-1.54 (-3.44, 0.36)	-1.03 (-2.29, 0.22)	-1.66 (-3.39, 0.07)	-2.10 (-4.48, 0.28)	-0.96 (-1.93, 0.01)
	Rate (kg/week)	-0.00 (-0.05, 0.04)	-0.03 (-0.08, 0.01)	**-0.05 (-0.11, -0.00)**	-0.06 (-0.14, 0.01)	-0.03 (-0.06, 0.00)
**Mid/Late**	*Unadjusted*	-1.46 (-3.05, 0.12)	-0.62 (-1.74, 0.51)	**-1.69 (-3.11, -0.27)**	-1.00 (-3.03, 1.02)	**-0.88 (-1.74, -0.03)**
	Minimally Adjusted	**-1.85 (-3.57, -0.14)**	-1.15 (-2.48, 0.17)	**-2.03 (-3.57, -0.48)**	-1.55 (-3.80, 0.69)	**-1.07 (-1.97, -0.17)**
	Fully Adjusted	-1.25 (-2.94, 0.43)	-0.59 (-1.91, 0.72)	-1.50 (-3.03, 0.03)	-1.58 (-3.70, 0.53)	**-0.90 (-1.76, -0.04)**
	Rate (kg/week)	-0.04 (-0.11, 0.02)	-0.02 (-0.07, 0.03)	-0.05 (-1.13, 0.01)	-0.06 (-0.01, 0.02)	**-0.04 (-0.07, -0.00)**
**3rd Tri**	*Unadjusted*	-0.76 (-1.78, 0.26)	-0.10 (-0.82, 0.63)	-0.47 (-1.39, 0.46)	-0.99 (-2.28, 0.29)	-0.32 (-0.87, 0.24)
	Minimally Adjusted	-0.93 (-2.02, 0.16)	-0.30 (-1.15, 0.55)	-0.65 (-1.65, 0.35)	-1.39 (-2.80, 0.02)	-0.36 (-0.94, 0.22)
	Fully Adjusted	-0.74 (-1.83, 0.35)	-0.12 (-0.97, 0.73)	-0.49 (-1.50, 0.52)	**-1.43 (-2.82, -0.04)**	-0.30 (-0.87, 0.28)
	Rate (kg/week)	-0.03 (-0.13, 0.06)	0.02 (-0.06, 0.09)	-0.02 (-0.10, 0.07)	-0.12 (-0.24, 0.00)	-0.02 (-0.07, 0.02)
**2nd Tri**	*Unadjusted*	-0.70 (-1.71, 0.31)	-0.52 (-1.23, 0.18)	**-1.22 (-2.11, -0.33)**	-0.01 (-1.29, 1.27)	**-0.57 (-1.11, -0.03)**
	Minimally Adjusted	-0.92 (-1.99, 0.14)	**-0.85 (-1.67, -0.04)**	**-1.38 (-2.32, -0.43)**	-0.17 (-1.57, 1.23)	**-0.71 (-1.26, -0.16)**
	Fully Adjusted	-0.64 (-1.67, 0.39)	-0.60 (-1.39, 0.19)	**-1.11 (-2.03, -0.18)**	-0.15 (-1.48, 1.18)	**-0.61 (-1.14, -0.08)**
	Rate (kg/week)	-0.05 (-0.12, 0.03)	-0.04 (-0.10, 0.01)	**-0.08 (-0.15, -0.01)**	-0.01 (-0.11, 0.08)	**-0.04 (-0.08, -0.01)**
**BMI>25 kg/m^2 (n=129)**						
**Total**	*Unadjusted*	2.18 (-0.14, 4.49)	0.35 (-1.67, 2.38)	1.77 (-0.28, 3.81)	0.25 (-2.51, 3.01)	**1.85 (0.43, 3.28)**
	Minimally Adjusted	1.18 (-0.99, 3.35)	-1.75 (-3.78, 0.29)	1.17 (-0.72, 3.05)	-1.68 (-4.35, 1.00)	1.22 (-0.11, 2.54)
	Fully Adjusted	1.10 (-1.03, 3.24)	**-2.16 (-4.17, -0.15)**	0.82 (-1.06, 2.70)	-1.78 (-4.43, 0.87)	1.17 (-0.14, 2.47)
	Rate (kg/week)	0.03 (-0.04, 0.01)	**-0.07 (-0.14, -0.01)**	0.02 (-0.04, 0.08)	-0.05 (-0.13, 0.03)	0.03 (-0.01, 0.07)
**Mid/Late**	*Unadjusted*	1.37 (-0.62, 3.35)	-0.08 (-1.81, 1.65)	1.04 (-0.71, 2.80)	0.10 (-2.25, 2.45)	**1.31 (0.09, 2.53)**
	Minimally Adjusted	0.58 (-1.28, 2.44)	**-1.83 (-3.56, -0.10)**	0.55 (-1.07, 2.16)	-1.51 (-3.80, 0.77)	0.78 (-0.36, 1.92)
	Fully Adjusted	0.51 (-1.31, 2.33)	**-2.13 (-3.83, -0.43)**	0.28 (-1.33, 1.88)	-1.50 (-3.75, 0.76)	0.71 (-0.41, 1.83)
	Rate (kg/week)	0.02 (-0.06, 0.09)	**-0.09 (-0.15, -0.02)**	0.01 (-0.06, 0.07)	0.06 (-0.15, 0.03)	0.03 (-0.02, 0.07)
**3rd Tri**	*Unadjusted*	0.46 (-0.71, 1.64)	-0.22 (-1.23, 0.80)	0.36 (-0.68, 1.39)	-0.15 (-1.54, 1.23)	0.65 (-0.08, 1.37)
	Minimally Adjusted	0.16 (-1.00, 1.32)	-0.98 (-2.06, 0.11)	0.15 (-0.86, 1.16)	-0.80 (-2.23, 0.63)	0.52 (-0.19, 1.23)
	Fully Adjusted	0.10 (-1.05, 1.24)	**-1.16 (-2.23, -0.09)**	0.02 (-0.99, 1.03)	-0.87 (-2.27, 0.53)	0.49 (-0.21, 1.19)
	Rate (kg/week)	0.01 (-0.10, 0.12)	-0.10 (-0.20, 0.00)	-0.01 (-0.10, 0.09)	-0.05 (-0.18, 0.08)	0.05 (-0.02, 0.11)
**2nd Tri**	*Unadjusted*	0.90 (-0.23, 2.04)	0.14 (-0.85, 1.12)	0.68 (-0.32, 1.68)	0.25 (-1.09, 1.60)	0.66 (-0.04, 1.36)
	Minimally Adjusted	0.42 (0.61, 1.44)	-0.85 (-1.81, 0.10)	0.39 (-0.49, 1.28)	0.71 (-1.97, 0.55)	0.26 (-0.37, 0.89)
	Fully Adjusted	0.38 (-0.62, 1.39)	**-0.99 (-1.94, -0.04)**	0.26 (-0.63, 1.15)	-0.72 (-1.96, 0.51)	0.22 (-0.40, 0.84)
	Rate (kg/week)	0.03 (-0.04, 0.10)	-0.07 (-0.14, 0.00)	0.02 (-0.04, 0.08)	-0.05 (-0.14, 0.04)	0.02 (-0.03, 0.06)

Abbr: BMI**:** Body Mass Index**,** GWG: Gestational Weight Gain, IQR: Interquartile Range, PFAS: Mid/Late: refers to 2nd+3rd trimester weight gain. PFAS:Perflouralkyl Substances, PFDA: perfluorodecanoic acid; PFHxS: perfluorohexanesulfonic acid, PFNA: perfluorononanoic acid; PFOA: perfluorooctanoic acid; PFOS: perfluorooctanesulfonic acid, Tri: Trimester.

† Minimally adjusted models include these covariates : maternal race/ethnicity, education, parity, age, early pregnancy BMI, and smoking. Fully adjusted model for trimester 2, trimester 3, and rate (kg/week) models includes minimally adjusted model covariates and: PFAS serum weeks, mid-late pregnancy kcal/day and METs/week. Fully adjusted model for mid/late and total GWG additionally includes gestational age at delivery.

*Bolded values indicate $p<0.05$

Supplementary Table 6. Multivariable linear models examining per interquartile range increase in log-transformed PFAS (ng/ml) in relation to mid/late gestational weight gain (kilograms) in the overall UPSIDE cohort and stratified by lower (BMI < 25 kg/m^2) versus higher (BMI>=25 kg/m^2) early pregnancy BMI†.

Model	PFOS ß (95% CI)	PFOA ß (95% CI)	PFNA ß (95% CI)	PFHxS ß (95% CI)	PFDA ß (95% CI)
All participants (n=243)_					
IQR	0.57	0.74	0.67	0.53	1.14
Per IQR	-0.23 (-2.44, 2.00)	**-1.89 (-3.34, -0.45)**	-0.73 (-2.39, 0.93)	-2.53 (-5.45, 0.40)	0.04 (-0.60, 0.67)
**BMI<25 kg/m^2 (n=114)**					
IQR	0.51	0.85	0.72	0.54	1.02
Per IQR	-2.45 (-5.76, 0.84)	-0.69 (-2.25, 0.85)	-2.08 (-4.21, 0.04)	-2.93 (-6.85, 0.98)	**-0.88 (-7.61, -0.04)**
**BMI>25 kg/m^2 (n=129)**					
IQR	0.58	0.65	0.59	0.55	1.12
Per IQR	0.88 (-2.26, 4.02)	**-3.28 (-5.89, -0.66)**	0.47 (-2.25, 3.19)	-2.73 (-6.82, 1.38)	0.63 (-0.37, 1.63)

Abbr: BMI**:** Body Mass Index**,** GWG: Gestational Weight Gain, IQR: Interquartile Range, PFAS: Mid/Late: refers to 2nd+3rd trimester weight gain. PFAS:Perflouralkyl Substances, PFDA: perfluorodecanoic acid; PFHxS: perfluorohexanesulfonic acid, PFNA: perfluorononanoic acid; PFOA: perfluorooctanoic acid; PFOS: perfluorooctanesulfonic acid, Tri: Trimester.

† Model includes these covariates : maternal race/ethnicity, education, parity, age, early pregnancy BMI, and smoking, PFAS serum weeks, mid-late pregnancy kcal/day, METs/week and gestational age at delivery.

*Bolded values indicate p<0.05

Supplementary Table 7. Multivariable linear models examining second trimester log-transformed PFAS (ng/ml) in relation to total \gestational weight gain (kg) in the overall UPSIDE cohort and stratified by lower (BMI < 25 kg/m²) versus higher (BMI>=25 kg/m²) early pregnancy BMI, excluding women who went on to deliver preterm (<37 weeks gestation; n=13) †,*.

	All All (N=230)		Pre-pregnancy BMI <25 kg/m² (N=110)		Pre-pregnancy BMI >=25 kg/m² (N=120)	
	**IQR**	**ß (95% CI)**	**IQR**	**ß (95% CI)**	**IQR**	**ß (95% CI)**
**PFOS**	0.56	0.24 (-1.29, 1.76)	0.49	-1.87 (-3.80, 0.06)	0.58	1.53 (-0.70, 3.76)
Per IQR		0.43 (-2.30, 3.14)		-3.82 (-7.76, 0.12)		2.64 (-1.21, 6.48)
**PFOA**	0.73	**-1.49 (-2.76, -0.19)**	0.75	-0.63 (-2.10, 0.84)	0.63	**-2.16 (-4.27, -0.04)**
Per IQR		**-2.04 (-3.78, -0.26)**		-0.84 (-280, 1.12)		**-3.43 (-6.78, -0.06)**
**PFNA**	0.64	-0.19 (-1.51, 1.14)	0.70	**-1.73 (-3.45, -0.02)**	0.56	1.10 (-0.87, 3.08)
Per IQR		-0.30 (-2.36, 1.78)		**-2.47 (-4.93, -0.03)**		1.96 (-1.55, 5.50)
**PFHxS**	0.53	-1.62 (-3.47, 0.22)	0.54	-2.38 (-4.77, 0.02)	0.56	-1.51 (-4.25, 1.23)
Per IQR		-3.06 (-6.55, 0.42)		-4.41 (-8.83, 0.04)		-2.70 (-7.59, 2.20)
**PFDA**	1.13	0.24 (-0.62, 1.11)	1.01	-0.85 (-1.81, 0.11)	1.05	1.34 (-0.06, 2.75)
Per IQR		0.21 (-0.55, 0.98)		-0.84 (-1.79, 0.11)		1.28 (-0.06, 2.62)

Abbr: BMI**:** Body Mass Index**,** GWG: Gestational Weight Gain, PFAS: Perflouralkyl Substances, PFDA: perfluorodecanoic acid; PFHxS: perfluorohexanesulfonic acid, PFNA: perfluorononanoic acid; PFOA: perfluorooctanoic acid; PFOS: perfluorooctanesulfonic acid, Tri: Trimester.

† Models adjusted for PFAS serum weeks, smoking, parity, maternal age, early pregnancy BMI, education, race/ethnicity, mid-late pregnancy kcal/day, METs/week, gestational age at delivery.

*Bolded values indicate $p<0.05$

Supplementary Table 8. p-value for interaction term (PFAS*pre-pregnancy BMI) in multivariable linear regression models examining log-transformed PFAS (ng/ml) in relation to total gestational weight gain (GWG; in kgs), post-partum weight retention (PPWR; in kg and body fat percentage at 6 and 12 months in the UPSIDE cohort.

	Pregnancy (n=243)	6 months post-partum (n=124)		12 months post-partum (n=104)	
	Total GWG†	PPWR‡	Body Fat %‡	PPWR‡	Body Fat %‡
**PFOS**	0.03	0.94	0.65	0.72	0.29
**PFOA**	0.47	0.43	0.77	0.15	0.51
**PFNA**	0.09	0.70	0.33	0.66	0.27
**PFHXS**	0.97	0.83	0.89	0.41	0.26
**PFDA**	0.08	0.76	0.16	0.66	0.12

* indicates p<0.05.

Abbr: BMI: Body Mass Index; GWG: Gestational Weight Gain, PFAS: per- and poly-fluoroalkyl substances, PFDA: perfluorodecanoic acid; PFHxS: perfluorohexanesulfonic acid, PFNA: perfluorononanoic acid; PFOA: perfluorooctanoic acid; PFOS: perfluorooctanesulfonic acid; Tri: trimester.

† Models adjusted for maternal age, early pregnancy BMI, parity, gestational age at delivery, gestational age at PFAS sampling, smoking, race/ethnicity, energy intake (kcal/day), physical activity (mets/week).

‡ Models adjusted for race/ethnicity, education, maternal age, parity, pre-pregnancy BMI, energy intake (kcal/day at 6m), physical activity (METs/day at 6 or 12 months), gestational age at PFAS sampling, gestational age at delivery, breastfeeding (yes/no) and weeks post-partum as covariates. * indicates p<0.05.

Supplementary Table 9. Spearman-rank correlation between log transformed PFAS (ng/ml) and post-partum weight retention (PPWR, in kg) and body fat percentage in the UPSIDE cohort

Visit	Group	Outcome	PFOS	PFOA	PFNA	PFHxS	PFDA
**6 Months postpartum**	**All (n=124)**	*PPWR*	-0.17	-0.20*	-0.10	-0.23**	-0.10
		Body Fat %	-0.09	-0.14	-0.08	-0.19*	-0.22*
	**BMI<25 kg/m^2 (n=66)**	*PPWR*	-0.16	-0.11	-0.19	-0.10	-0.13
		Body Fat %	0.003	-0.05	-0.17	0.02	-0.14
	**BMI>25 kg/m^2 (n=58)**	*PPWR*	-0.15	-0.24	-0.06	-0.28*	-0.02
		Body Fat %	0.02	-0.16	-0.05	-0.20	-0.09
**12 months postpartum**	**All (n=104)**	*PPWR*	-0.30	-0.25**	-0.17	-0.32***	-0.10
		Body Fat %	-0.18	-0.14	-0.06	-0.31**	-0.21*
	**BMI<25 kg/m^2 (n=52)**	*PPWR*	-0.21	-0.02	-0.15	-0.26	-0.09
		Body Fat %	-0.01	0.07	-0.12	-0.05	-0.09
	**BMI>25 kg/m^2 (n=52)**	*PPWR*	-0.32*	-0.36**	-0.21	-0.30*	-0.05
		Body Fat %	-0.26	-0.30*	-0.19	-0.27	-0.15

Abbr: BMI: Body Mass Index; GWG: PFAS: per- and poly-fluoroalkyl substances, PFDA: perfluorodecanoic acid; PFHxS: perfluorohexanesulfonic acid, PFNA: perfluorononanoic acid; PFOA: perfluorooctanoic acid; PFOS: perfluorooctanesulfonic acid; PPWR: post -partum weight retention.
* indicates p<0.05.** indicates p<0.01. *** indicates p<0.001.

Supplementary Table 10. **Unadjusted and adjusted linear models examining second trimester log-transformed PFAS (ng/ml) in relation to post-partum weight retention (PPWR) in kg and total body fat percentage at 6 and 12 months post-delivery in the UPSIDE cohort†,*.**

PFAS	Model	All	BMI <25 kg/m^2	BMI>25 kg/m^2
**6 months postpartum**				
		**n=124; β (95%CI)**	**n=66; β (95%CI)**	**n=58; β (95%CI)**
PPWR				
PFOS	Unadjusted	-1.92 (-4.05, 0.22)	-1.20 (-3.44, 1.03)	-2.42 (-6.36, 1.51)
	Minimally adjusted	-1.76 (-3.85, 0.32)	-1.33 (-3.56, 0.91)	**-2.40 (-6.50, 1.70)**
	Fully adjusted	-2.15 (-4.43, 0.13)	-1.31 (-3.80, 1.18)	**-5.50 (-9.84, -1.17)**
PFOA	Unadjusted	-1.44 (-2.94, 0.05)	-0.63 (-2.26, 1.00)	-2.08 (-4.72, 0.56)
	Minimally adjusted	**-2.10 (-3.65, -0.56)**	-0.53 (-2.53, 1.46)	**-3.12 (-5.58, -0.66)**
	Fully adjusted	**-2.39 (-4.17, -0.61)**	-0.64 (-2.79, 1.50)	**-4.27 (-7.22, -1.31)**
PFNA	Unadjusted	-0.57 (-2.23, 1.09)	-1.26 (-3.22, 0.69)	-0.11 (-2.82, 2.60)
	Minimally adjusted	-0.81 (-2.33, 0.72)	-1.27 (-3.28, 0.74)	-0.41 (-2.88, 2.05)
	Fully adjusted	-0.78 (-2.51, 0.96)	-1.32 (-3.72, 1.08)	-1.42 (-4.25, 1.40)
PFHxS	Unadjusted	**-3.56 (-5.98, -1.15)**	-1.27 (-4.17, 1.63)	**-4.84 (-8.87, -0.82)**
	Minimally adjusted	**-2.57 (-5.03, -0.11)**	-0.44 (-3.54, 2.67)	**-4.22 (-8.40, -0.06)**
	Fully adjusted	**-2.94 (-5.52, -0.35)**	-0.71 (-3.80, 2.39)	**-5.00 (-9.43, -0.58)**
PFDA	Unadjusted	-0.31 (-1.45, 0.83)	-0.47 (-1.68, 0.75)	0.40 (-1.76, 2.56)
	Minimally adjusted	-0.17 (-1.22, 0.88)	-0.52 (-1.72, 0.68)	0.38 (-1.52, 2.29)
	Fully adjusted	0.10 (-1.11, 1.31)	0.07 (-1.28, 1.43)	0.42 (-2.00, 2.85)
**Body Fat %**				
PFOS	Unadjusted	-1.50 (-4.59, 1.60)	0.04 (-2.93, 3.02)	0.22 (-3.67, 4.11)
	Minimally adjusted	-0.65 (-2.49, 1.18)	-0.37 (-2.90, 2.16)	-0.89 (-3.47, 1.69)
	Fully adjusted	-0.33 (-2.25, 1.60)	-0.11 (-2.88, 2.67)	-1.08 (-3.85, 1.69)
PFOA	Unadjusted	-1.63 (-3.78, 0.51)	-0.46 (-2.76, 1.83)	-1.44 (-3.82, 0.94)
	Minimally adjusted	**-1.60 (-2.95, -0.24)**	-1.74 (-3.93, 0.46)	-1.53 (-3.10, 0.04)
	Fully adjusted	**-1.75 (-3.17, -0.32)**	-1.87 (-4.30, 0.57)	-1.64 (-3.41, 0.13)
PFNA	Unadjusted	-1.07 (-3.43, 1.30)	-1.82 (-4.43, 0.78)	-0.47 (-3.01, 2.07)
	Minimally adjusted	-0.97 (-2.30, 0.35)	-1.67 (-3.91, 0.56)	-0.53 (-2.06, 1.00)
	Fully adjusted	-0.79 (-2.19, 0.62)	-1.53 (-4.16, 1.10)	-0.62 (-2.29, 1.05)
PFHxS	Unadjusted	**-4.04 (-7.70, -0.38)**	0.35 (-3.76, 4.46)	-3.07 (-7.12, 0.98)
	Minimally adjusted	-1.47 (-3.64, 0.70)	0.21 (-3.26, 3.69)	**-3.18 (-5.73, -0.63)**
	Fully adjusted	-1.44 (-3.65, 0.77)	0.10 (-3.52, 3.71)	**-3.17 (-5.85, -0.50)**
PFDA	Unadjusted	**-2.00 (-3.56, -0.44)**	-0.94 (-2.65, 0.68)	-0.66 (-2.56, 1.25)
	Minimally adjusted	-0.30 (-1.21, 0.62)	-0.86 (-2.19, 0.47)	0.19 (-1.00, 1.38)
	Fully adjusted	-0.08 (-1.04, 0.87)	-0.47 (-2.00, 1.06)	0.28 (-1.02, 1.59)
**12 months postpartum**				
**PPWR**				
		**N=104; β (95%CI)**	**N=52; β (95%CI)**	**N=52; β (95%CI)**
PFOS	Unadjusted	**-3.74 (-6.25, -1.23)**	-2.02 (-4.90, 0.86)	**-4.58 (-8.52, -0.65)**
	Minimally adjusted	**-3.55 (-6.10, -1.02)**	-2.46 (-5.27, 0.36)	**-5.64 (-9.94, -1.44)**
	Fully adjusted	**-4.08 (-6.78, -1.38)**	-2.78 (-6.20, 0.64)	**-6.28 (-10.85, -1.70)**
PFOA	Unadjusted	**-2.86 (-5.15, -0.58)**	-0.07 (-2.49, 2.34)	**-4.97 (-8.68, -1.25)**
	Minimally adjusted	**-3.35 (-5.64, -1.06)**	-0.97 (-3.71, 1.76)	**-5.02 (-8.61, -1.44)**
	Fully adjusted	**-4.02 (-6.58, -1.46)**	-1.48 (-4.69, 1.72)	**-7.39 (-11.49, -3.29)**
PFNA	Unadjusted	-1.54 (-3.76, 0.68)	-1.27 (-4.00, 1.45)	-2.08 (-5.44, 1.29)
	Minimally adjusted	-1.64 (-3.67, 0.40)	-2.01 (-4.71, 0.70)	-1.99 (-5.11, 1.14)
	Fully adjusted	-1.94 (-4.24, 0.36)	-2.26 (-5.62, 1.09)	-3.11 (-6.87, 0.66)
PFHxS	Unadjusted	**-5.38 (-8.31, -2.45)**	-3.43 (-6.92, 0.06)	**-5.70 (-10.56, -0.84)**
	Minimally adjusted	**-4.62 (-7.72, -1.53)**	-3.16 (-6.80, 0.49)	**-5.50 (-10.56, -0.43)**
	Fully adjusted	**-5.13 (-8.34, -1.93)**	-3.21 (-7.14, 0.73)	**-6.53 (-12.01, -1.06)**
PFDA	Unadjusted	-0.42 (-1.90, 1.07)	-0.37 (-1.96, 1.22)	0.04 (-2.46, 2.53)
	Minimally adjusted	-0.23 (-1.63, 1.17)	-0.63 (-2.21, 0.95)	-0.17 (-2.56, 2.22)
	Fully adjusted	-0.19 (-1.85, 1.46)	-0.61 (-2.49, 1.27)	-0.05 (-3.29, 3.18)
**Body Fat %**				
PFOS	Unadjusted	-3.13 (-6.42, 0.16)	-0.15 (-3.32, 3.02)	-3.10 (-6.41, 0.20)
	Minimally adjusted	-0.26 (-2.20, 1.68)	0.56 (-1.99, 3.11)	-1.55 (-4.08, 0.98)
	Fully adjusted	-0.14 (-2.15, 1.87)	0.37 (-2.18, 2.92)	-1.69 (-4.48, 1.10)
PFOA	Unadjusted	-2.02 (-4.90, 0.86)	0.67 (-2.17, 3.50)	**-3.06 (-5.81, -0.31)**
	Minimally adjusted	-1.42 (-3.15, 0.31)	-0.73 (-3.13, 1.67)	-1.96 (-4.08, 0.17)
	Fully adjusted	-1.64 (-3.43, 0.16)	-1.05 (-3.60, 1.50)	-2.15 (-4.44, 0.14)
PFNA	Unadjusted	-0.82 (-3.60, 1.96)	-1.29 (-4.25, 1.67)	-1.72 (-4.31, 0.87)
	Minimally adjusted	-0.24 (-1.76, 1.27)	-0.89, -3.31, 1.52)	-0.59 (-2.39, 1.21)
	Fully adjusted	-0.27 (-1.86, 1.33)	-0.71 (-3.15, 1.72)	-0.75 (-2.80, 1.31)
PFHxS	Unadjusted	**-6.59 (-10.52, -2.66)**	-0.79 (-5.06, 3.48)	-4.00 (-8.02, 0.03)
	Minimally adjusted	-1.99 (-4.33, 0.35)	0.59 (-2.71, 3.90)	**-3.18 (-6.05, -0.32)**
	Fully adjusted	-1.92 (-4.32, 0.47)	0.88 (-2.33, 4.09)	**-3.39 (-6.49, -0.29)**
PFDA	Unadjusted	**-1.96 (-3.76, -0.17)**	-0.54 (-2.30, 1.22)	-1.01 (-2.87, 0.84)
	Minimally adjusted	0.21 (-0.82, 1.24)	-0.42 (-1.81, 0.97)	0.01 (-1.35, 1.37)

	Fully adjusted	0.23 (-0.87, 1.34)	-0.12 (-1.53, 1.29)	0.16 (-1.41, 1.74)

Abbr: BMI: Body Mass Index; PFAS: per- and poly-fluoroalkyl substances, PFDA: perfluorodecanoic acid; PFHxS: perfluorohexanesulfonic acid, PFNA: perfluorononanoic acid; PFOA: perfluorooctanoic acid; PFOS: perfluorooctanesulfonic acid.

† Minimally adjusted models include these covariates: race/ethnicity, education, maternal age, parity, pre-pregnancy BMI. Fully adjusted models include minimally adjusted model covariates and energy intake (kcal/day at 6m), physical activity (METs/week at 6 or 12 months), gestational age at PFAS sampling, gestational age at delivery, breastfeeding (yes/no) and weeks post-partum as covariates.

Supplementary Table 11. Multivariable linear models examining per interquartile range increase in log-transformed PFAS (ng/ml) in relation to post-partum weight retention (PPWR) in kg and total body fat percentage at 6 and 12 months post-delivery in the UPSIDE cohort in the overall UPSIDE cohort and stratified by lower (BMI < 25 kg/m^2) versus higher (BMI>=25 kg/m^2) early pregnancy BMI†.

Model	**PFOS** ß (95% CI)	**PFOA** ß (95% CI)	**PFNA** ß (95% CI)	**PFHxS** ß (95% CI)	**PFDA** ß (95% CI)
6 months postpartum					
**All participants (n=124)_**					
IQR	0.49	**0.74**	0.68	0.57	1.24
PPWR	-4.39 (-9.04, 0.27)	**-3.23 (-5.64, -0.82)**	-1.15 (-3.69, 1.41)	**-5.16 (-9.68, -0.61)**	0.08 (-0.90, 1.06)
Body fat %	-0.67 (-4.59, 3.27)	**-2.36 (-4.28, -0.43)**	-1.16 (-3.22, 0.91)	-2.53 (-6.40, 1.35)	-0.06 (-0.84, 0.70)
**BMI<25 kg/m^2 (n=66)**					
IQR	0.62	0.85	0.78	0.53	0.97
PPWR	-2.11 (-6.13, 1.90)	-0.75 (-3.28, 1.76)	-1.69 (-4.77, 1.38)	-1.34 (-7.17, 4.51)	0.07 (-1.32, 1.47)
Body fat %	-0.18 (-4.65, 4.31)	-2.20 (-5.06, 0.67)	-1.96 (-5.33, 1.41)	0.19 (-6.64, 7.00)	-0.48 (-2.06, 1.09)
**BMI>25 kg/m^2 (n=58)**					
IQR	0.46	0.67	0.53	0.58	1.55
PPWR	**-11.96 (-21.39, -2.54)**	**-6.37 (-10.78,-1.96)**	-2.68 (-8.02, 2.64)	**-8.62 (-16.26, -1.00)**	0.27 (-1.29, 1.84)
Body fat %	-2.35 (-8.37, 3.67)	**-2.45 (-5.09, 0.19)**	-1.17 (-4.32, 1.98)	**-5.47 (-10.09, -0.86)**	0.18 (-0.66, 1.03)
12 months postpartum **(n=104)**					
IQR	**0.52**	0.68	0.72	0.55	1.21
PPWR	**-7.85 (-13.04, -2.65)**	**-5.91 (9.68, -2.15)**	-2.69 (-5.89, 0.50)	**-9.33 (-15.16, -3.51)**	-0.16 (-1.53, 1.21)
Body fat %	-0.27 (-4.13, 3.60)	-2.41 (-5.04, 0.24)	-0.38 (-2.58, 1.85)	-3.49 (-7.85, 0.85)	0.19 (-0.72, 1.11)
**BMI<25 kg/m^2 (n=52)**					
IQR	0.57	0.76	0.76	0.46	0.98
PPWR	-4.88 (-10.88, 1.12)	-1.95 (-6.17, 2.26)	-2.97 (-7.39, 1.43)	-6.98 (-15.52, 1.59)	-0.62 (-2.54, 1.30)
Body fat %	0.65 (-3.82, 5.12)	-1.38 (-4.74, 1.97)	-0.93 (-4.14, 2.26)	1.91 (-5.07, 8.89)	-0.12 (-1.56, 1.32)
**BMI>25 kg/m^2 (n=52)**					
IQR	0.51	**0.61**	0.61	0.62	1.46
PPWR	**-12.31 (-21.27, -3.33)**	**-12.11 (-18.84, -5.39)**	-5.10 (-11.26, 1.08)	**-10.53 (-19.37, -1.71)**	-0.03 (-2.25, 2.18)
Body fat %	-3.31 (-8.78, 2.16)	**-3.52 (-7.28, 0.23)**	-1.23 (-4.59, 2.15)	**-5.47 (-10.47, -0.47)**	0.11 (-0.97, 1.19)

Abbr: BMI**:** Body Mass Index**,** GWG: Gestational Weight Gain, IQR: Interquartile Range, PFAS: Mid/Late: refers to 2nd+3rd trimester weight gain. PFAS:Perflouralkyl Substances, PFDA: perfluorodecanoic acid; PFHxS: perfluorohexanesulfonic acid, PFNA: perfluorononanoic acid; PFOA: perfluorooctanoic acid; PFOS: perfluorooctanesulfonic acid.
† Model includes these covariates : maternal race/ethnicity, education, parity, age, early pregnancy BMI, and smoking, PFAS serum weeks, mid-late pregnancy kcal/day, METs/week and gestational age at delivery.
*Bolded values indicate p<0.05

Supplementary Table 12. Association between weighted quantile sum* of PFAS exposure and prenatal and postpartum maternal weight outcomes in UPSIDE/UPSIDE-Moms cohort.

	**All Participants**	**BMI<25 kg/m^2**	**BMI>25 kg/m^2**
	WQS Beta (95% CI)	WQS Beta (95% CI)	WQS Beta (95% CI)
Prenatal †			
n	243	114	129
Mid/Late GWG Rate			
Minimally adjusted (negative)	0.0016 (-0.027, 0.030)	-0.034 (-0.070, 0.002)	0.003 (-0.048, 0.053)
Fully adjusted (negative)	0.003 (-0.026, 0.031)	-0.034 (-0.069, 0.001)	-0.001 (-0.048, 0.046)
Minimally adjusted (positive)	-0.016 (-0.040, 0.007)	**-0.045 (-0.079, -0.010)**	-0.003 (-0.056, 0.050)
Fully adjusted (positive)	-0.016 (-0.039, 0.008)	**-0.047 (-0.080, -0.013)**	-0.003 (-0.059, 0.053)
Postpartum ‡			
6 M Participant	124	66	58
Weight Retention			
Minimally adjusted (negative)	-0.0261 (-1.692, 1.169)	0.086 (-1.256, 1.428)	0.459 (-2.193, 3.112)
Fully adjusted (negative)	-0.127 (-1.674, 1.420)	0.909 (-0.357, 2.175)	-1.356 (-4.168, 1.455)
Minimally adjusted (positive)	0.367 (-0.675, 1.410) *	-0.167 (-1.491, 1.157) *	-0.036 (-2.792, 2.720)
Fully adjusted (positive)	0.328 (-0.745, 1.402) *	0.927 (-0.428, 2.281) *	-0.654 (-3.246, 1.938)
Body Fat %			
Minimally adjusted (negative)	-0.710 (-1.812, 0.391)	0.375 (-1.314, 2.064)	-0.152 (-2.077, 1.773)
Fully adjusted (negative)	-0.422 (-1.607, 0.762)	1.341 (-0.537, 3.220)	-1.235 (-3.334, 0.875)
Minimally adjusted (positive)	-0.420 (-1.609, 0.769)	0.488 (-1.026, 2.003) *	-0.312 (-1.973, 1.350) *
Fully adjusted (positive)	-0.011 (-1.397, 1.375)	0.703 (-1.158, 2.564)	-0.309 (-2.341, 1.723)
12 M Participants	104	52	52
Weight Retention			
Minimally adjusted (negative)	-1.523 (-3.101, 0.054)	-0.910 (-2.281, 0.462)	**-3.947 (-6.948, -0.947)**
Fully adjusted (negative)	**-2.030 (-3.486, -0.573)**	-1.423 (-3.073, 0.228)	**-4.920 (-8.657, -1.182)**
Minimally adjusted (positive)	-0.679 (-2.096, 0.739) *	-0.571 (-1.855, 0.712)	-3.461 (-6.968, 0.046)
Fully adjusted (positive)	**-1.789 (-3.482, -0.095)** *	-1.353 (-3.012, 0.306)	**-5.587 (-9.387, -1.787)**
Body Fat %			
Minimally adjusted (negative)	-0.542 (-1.767, 0.682)	-0.181 (-1.663, 1.300)	-1.532 (-3.486, 0.422) *
Fully adjusted (negative)	-0.749 (-2.063, 0.565)	0.902 (-0.731, 2.435)	**-2.788 (-5.460, -0.116)** *
Minimally adjusted (positive)	-0.405 (-1.699, 0.889)	-0.031 (-1.521, 1.460)	-1.934 (-4.-57, 0.189)
Fully adjusted (positive)	-0.613 (-1.970, 0.744)	0.766 (-0.887, 2.420)	**-2.914 (-5.498, -0.329)**

Abbr: BMI**:** Body Mass Index**,** GWG: Gestational Weight Gain, CI: Confidence Interval.
† Minimally adjusted models include these covariates : maternal race/ethnicity, education, parity, age, early pregnancy BMI, and smoking. Fully adjusted includes minimally adjusted covariates and: PFAS serum weeks, mid-late pregnancy kcal/day and METs/week, and gestational age at delivery.
‡ Minimally adjusted model is adjusted for race/ethnicity, education, maternal age, parity, pre-pregnancy BMI. Fully adjusted includes minimally adjusted covariates and energy intake (kcal/day at 6m), physical activity (METs/week at 6 or 12 months), gestational age at PFAS sampling, gestational age at delivery, breastfeeding (yes/no) and weeks post-partum as covariates.
* Estimates with less than 100 bootstraps are flagged. Bolded values indicate p<0.05.

Supplementary Table 13. Index weights for individual PFAS in WQS models† examining the association between PFAS and average rate of gain (kg/week) in mid/late pregnancy.

	Models with negative beta for WQS index			Models with positive beta for WQS index		
	All (n=243)	BMI <25 kg/m^2 (N=114)	BMI>25 kg/m^2 (N=129)	All (n=243)	BMI <25 kg/m^2 (N=114)	BMI>25 kg/m^2 (N=129)
**PFOS**	0.03	0.07	0.02	0.01	0.34	0.23
**PFOA**	0.26	0.33	0.77	0.00	0.12	0.00
**PFNA**	0.01	0.09	0.10	0.08	0.07	0.04
**PFHxS**	0.70	0.42	0.09	0.00	0.04	0.18
**PFDA**	0.00	0.09	0.02	0.91	0.42	0.54

Abbr: PFAS: per- and poly-fluoroalkyl substances, PFDA: perfluorodecanoic acid; PFHxS: perfluorohexanesulfonic acid, PFNA: perfluorononanoic acid; PFOA: perfluorooctanoic acid; PFOS: perfluorooctanesulfonic acid.

† Fully adjusted includes minimally adjusted covariates and energy intake (kcal/day at 6m), physical activity (METs/week at 6 or 12 months), gestational age at PFAS sampling, gestational age at delivery, breastfeeding (yes/no) and weeks post-partum as covariates.

Supplementary Figure 1. Histogram of number of clinically recorded weights per participant and chart of measurement timing of weights used for interpolation of weight at week 14 and 28

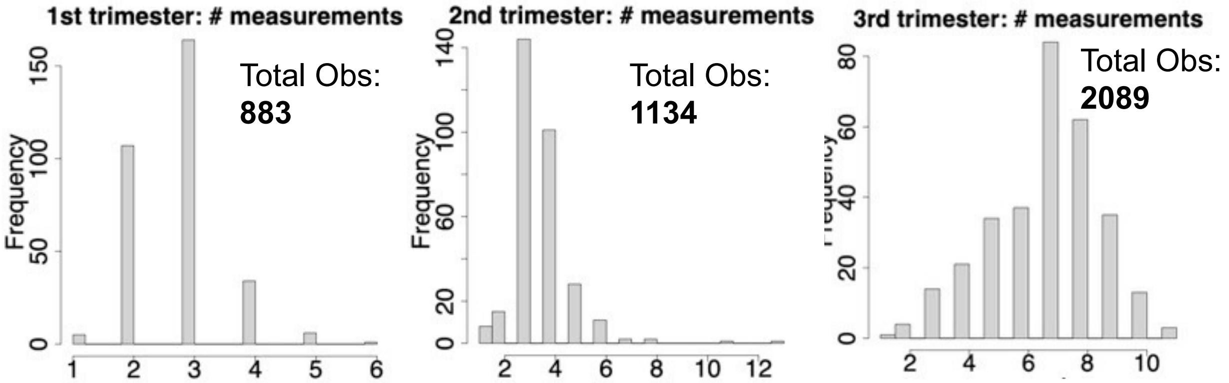

Mean (SD) gestational weeks of weights used for interpolation of weight at the end of trimester 1 and trimester 2			
Latest 1st Trimester	Earliest 2nd Trimester	Latest 2nd Trimester	Earliest 3rd Trimester
12.0 (1.4)	16.3 (1.5)	25.7 (2.2)	29.5 (1.1)

Abbreviations: Obs: observations; SD: standard deviation.

Supplementary Figure 2. Directed acyclic graphs

A. **PFAS and Gestational Weight Gain**

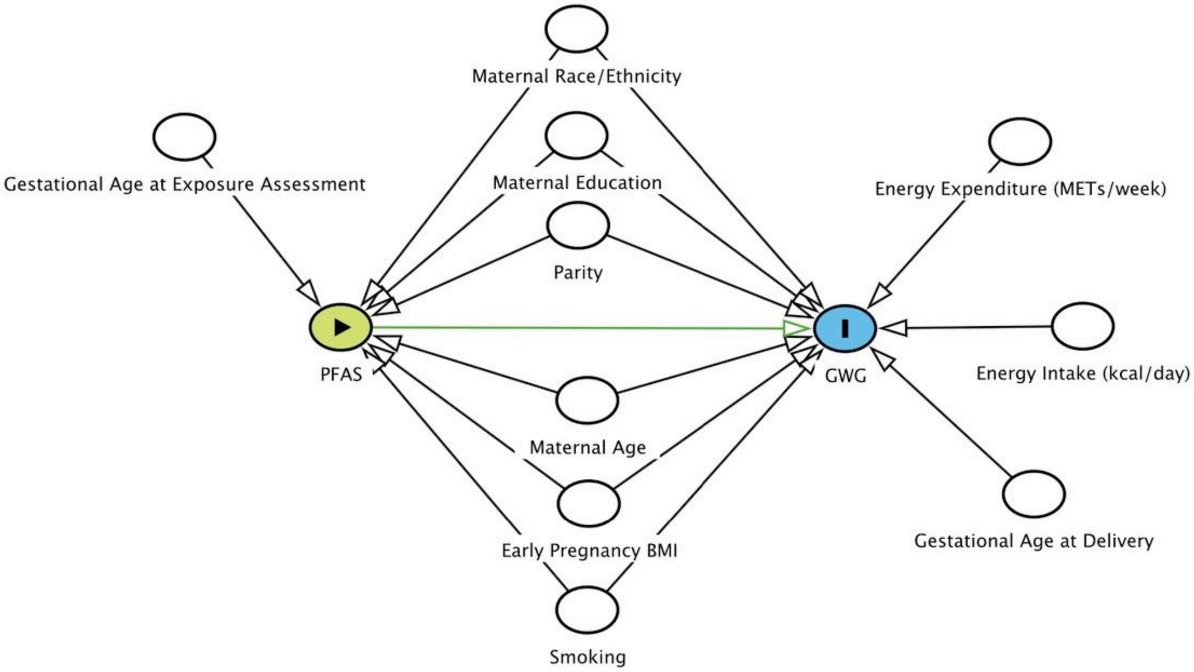

B. **PFAS and Postpartum Weight Retention and Body Fat**

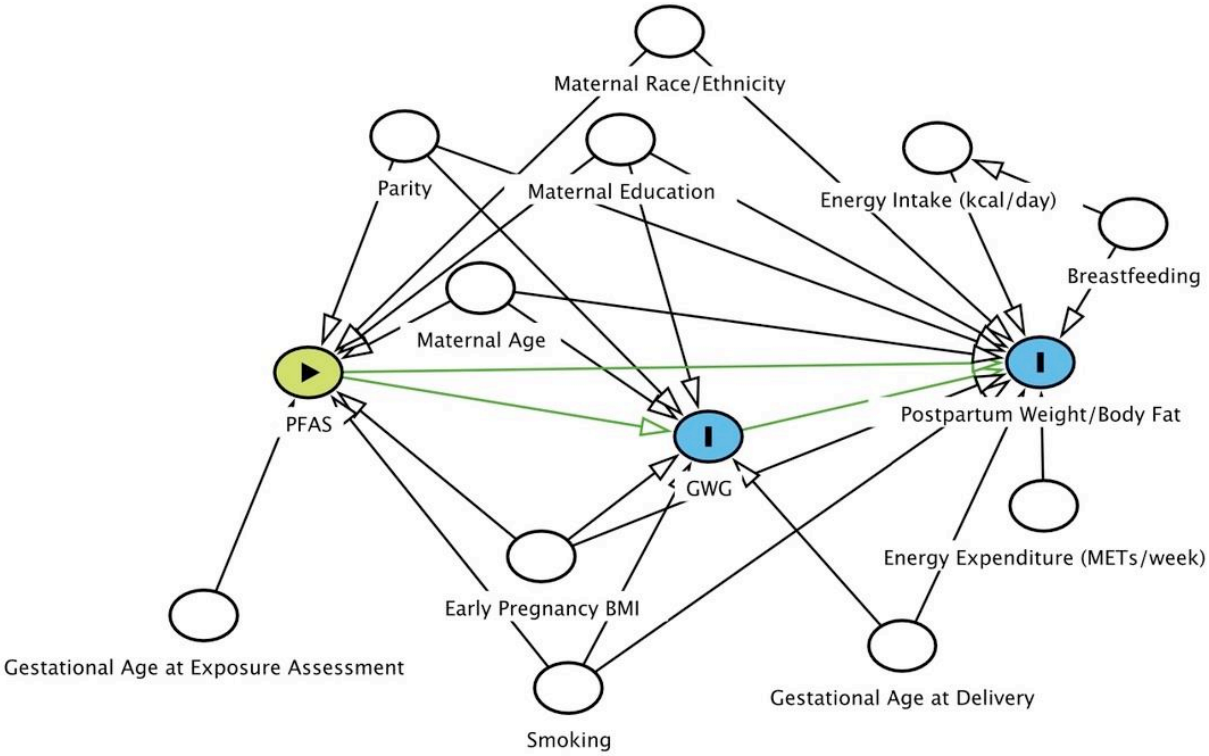

Supplement: Supplementary file 1 — Additional file 1: Supplementary Table 1. Summary of published epidemiological literature on prenatal PFAS concentrations and gestational weight gain and post-partum weight retention. Supplementary Figure 1. Histogram of number of clinically recorded weights per participant and table of measurement timing of weights used for weight interpolation. Supplementary Figure 2. Directed acyclic graph of covariates considered in the pre- and postnatal analysis. Supplementary Table 2. Descriptive Statistics of UPSIDE participants included in the current analysis and parent cohort. Supplementary Table 3. Spearman Correlation between log-transformed PFAS (ng/ml) in the UPSIDE cohort. Supplementary Table 4. Trimester specific – and total gestational weight gain by body mass index in the UPSIDE cohort. Supplementary Table 5. Multivariable linear models examining log-transformed PFAS (ng/ml) in relation to total and trimester-specific gestational weight gain (kg) in the overall UPSIDE cohort and stratified by lower (BMI < 25 kg/m2) versus higher (BMI>=25 kg/m2) early pregnancy BMI. Supplementary Table 6. Multivariable linear models examining per interquartile range increase in log-transformed PFAS (ng/ml) in relation to mid/late gestational weight gain (kilograms) in the overall UPSIDE cohort and stratified by lower (BMI < 25 kg/m2) versus higher (BMI>=25 kg/m2) early pregnancy BMI. Supplementary Table 7. Multivariable linear models examining second trimester log-transformed PFAS (ng/ml) in relation to total and trimester-specific gestational weight gain (kg) in the overall UPSIDE cohort and stratified by lower (BMI < 25 kg/m2) versus higher (BMI>=25 kg/m2) early pregnancy BMI, excluding women who went on to deliver preterm (<37 weeks gestation; n=13). Supplementary Table 8. p-value for interaction term (PFAS*pre-pregnancy BMI) in multivariable linear regression models examining log-transformed PFAS (ng/ml) in relation to total gestational weight gain (GWG; in kg), post-partum wei [file 12940_2023_1009_MOESM1_ESM.pdf]
